# Gut Microbiota Protects Against Liver Injury and Fibrosis via Activation of the CYP Eicosanoid Pathway

**DOI:** 10.21203/rs.3.rs-6356145/v1

**Published:** 2025-04-03

**Authors:** Guodong Zhang, Quancai Sun, Jing Sun, Nan Jing, Matthew Edin, Yige Wang, Weidong Xu, Jianan Zhang, Weicang Wang, Fred Lih, Weimin Wang, Vladimir Yeliseyev, Jie Lin, You-Tae Kim, Renhua Song, Sven Pettersson, Justin Wong, David Mills, Darryl Zeldin, Haixia Yang

## Abstract

Gut microbiota has been shown to play an important role in the pathogenesis of liver injury and fibrosis, but the specific microbial factors or pathways involved remain poorly defined. Here we show that specific gut microbial metabolites, notably indole, activate the cytochrome P450 (CYP) eicosanoid pathway in the liver and protect the liver against liver injury and fibrosis. Using LC-MS/MS-based lipidomics to compare conventionally raised mice with germ-free or antibiotic-treated mice, we show that the gut microbiota induces the CYP eicosanoid pathway in the liver. Furthermore, by administering exogenous indole or mono-colonizing germ-free mice with indole-producing Bacteroides thetaiotaomicron or a mutant strain lacking indole production, we demonstrate that gut bacteria-produced indole activates the liver’s CYP eicosanoid pathway through pregnane X receptor-dependent mechanisms. Finally, we find that disruption of the microbiota-liver-CYP axis, through inhibition or genetic ablation of CYP monooxygenases, or antibiotic suppression of gut microbiota, exacerbates chemically or surgically induced liver injury or fibrosis. Conversely, activation of this axis through indole administration attenuates liver injury and fibrosis. Together, these results demonstrate that the microbiota-liver-CYP axis plays a key role in the mechanisms by which gut microbiota interacts with the liver to influence host metabolism and disease progression.

Gut-liver axis, eicosanoids, liver injury, liver fibrosis, lipidomics, indole

## Introduction

Liver injury is a common pathological feature of many liver diseases, including drug-induced hepatotoxicity, alcoholic liver diseases, or viral hepatitis [1]. Prolonged liver injury can overwhelm the liver's repair and regeneration processes and lead to liver fibrosis, which is a major risk factor for developing life-threatening conditions such as liver cirrhosis and hepatocellular carcinoma [1,2]. Substantial studies support that gut microbiota, the trillions of bacteria inhabiting the gastrointestinal tract, plays an important role in the progression of liver injury and fibrosis [3]. Indeed, previous studies have consistently demonstrated that germ-free (GF) animals, established on different animal strains including ICR mice, Carworth Farms Swiss Webster mice, BALB/c mice, C57BL/6 mice, Wistar rats, and Fischer rats, exhibit an increased severity of liver injury or fibrosis that are induced by various chemical or surgical treatments, in comparison to their conventionally raised counterparts [4-10] (see summary of literature in **Table S1**). Overall, these findings support that the presence of gut microbiota protects the liver against liver injury and fibrosis [4-10]. However, the specific gut microbial factors or pathways responsible for the gut microbiota's hepatoprotective effects remain poorly defined, hampering our efforts to understand microbiota-liver interactions and develop therapeutic interventions for these liver disorders.

Eicosanoids refer to a group of endogenous lipid-based signaling molecules produced from the enzymatic metabolism of membrane phospholipid-incorporated polyunsaturated fatty acids [11,12]. There are three primary pathways involved in eicosanoid biosynthesis: the cyclooxygenase pathway (COX-1 and COX-2), which generates prostaglandins and thromboxanes; the lipoxygenase pathway (5-, 12-, and 15-LOX), responsible for producing leukotrienes and hydroxyl fatty acids; and the cytochrome P450 (CYP) monooxygenase pathway, which forms fatty acid epoxides and diols [11,12]. Eicosanoids have potent actions to regulate inflammation, immune responses, vascular tone, and tissue hemostasis, and are the therapeutic targets of many drugs on the market [11,12]. While previous research about the biosynthesis and function of eicosanoids has predominantly focused on host factors [11,12], the role of gut microbiota in these processes is unknown.

Given the importance of eicosanoid signaling in human health [11,12], we hypothesize that gut microbiota modulates the biosynthesis of tissue-protective eicosanoids in the liver, which subsequently protects the liver from injury and fibrosis. To test this hypothesis, we employed liquid chromatography-tandem mass spectrometry (LC-MS/MS)-based lipidomics to compare eicosanoid profiles in the livers of conventionally raised mice with those of GF or antibiotic-treated mice. We then assessed the roles of the microbiota-regulated eicosanoid pathway in the progression of liver injury and fibrosis. Using this strategy, herein we show that specific gut microbes, notably *Bacteroides thetaiotaomicron*, produce small-molecule metabolites such as indole. These microbial metabolites upregulate the liver's CYP eicosanoid pathway and protect the liver from liver injury and fibrosis.

## Results

### Gut microbiota induces the CYP eicosanoid pathway in the liver

To date, the impact of gut microbiota on host eicosanoid biosynthesis is unknown. We used LC-MS/MS-based lipidomics, which can measure 56 eicosanoids produced by COX, LOX, and CYP enzymes, to compare eicosanoid profiles in the liver of conventionally raised mice (specific pathogen-free or SPF mice) versus GF mice. We analyzed both free eicosanoids and total eicosanoids (a combination of free eicosanoids and membrane phospholipid-incorporated eicosanoid esters) in the liver (see the scheme of the experiment in **Fig. 1A**). LC-MS/MS identified 52 eicosanoids in the liver (see **Table S2-3** for complete LC-MS/MS results, some metabolites were below the detection limits of LC-MS/MS). For both free and total eicosanoids, the hepatic concentrations of a series of CYP eicosanoids are increased in SPF mice compared to GF mice (see volcano plot of liver eicosanoids in **Fig. 1B** and the biosynthetic pathway of CYP eicosanoids in **Fig. S1**). Indeed, the concentrations of CYP-produced fatty acid epoxides, including epoxyoctadecenoic acids (EpOMEs) derived from linoleic acid (LA, 18:2ω-6), epoxyeicosatrienoic acids (EETs) from arachidonic acid (ARA, 20:4ω-6), epoxyeicosatetraenoic acid (EpETEs) from eicosapentaenoic acid (EPA, 20:5ω-3), and epoxydocosapentaenoic acids (EpDPAs) from docosahexaenoic acid (DHA, 22:6ω-3) [12], are increased in the livers of SPF mice compared to those of GF mice (**Fig. 1C**, see structures of the metabolites in **Fig. S1**). Additionally, the concentrations of fatty acid diols, which are downstream metabolites of fatty acid epoxides produced by soluble epoxide hydrolase (sEH) [12], were also increased in the liver of SPF mice compared to GF mice (**Fig. 1D**, see structures of the metabolites in **Fig. S1**). The ratios of fatty acid diols to fatty acid epoxides, a surrogate marker for the function of sEH [12], were not significantly changed between SPF mice to GF mice (**Fig. S2**), suggesting that gut microbiota doesn't affect sEH-mediated lipid metabolism. Overall, these findings support the conclusion that gut microbiota elevates the hepatic concentrations of CYP monooxygenase-derived eicosanoids.

Since eicosanoids are usually locally produced and locally acting [11,12], we hypothesize that gut microbiota increases the liver's CYP eicosanoids by enhancing their biosynthesis in the liver. To test this, we examined the hepatic expression of Cyp monooxygenases (predominately Cyp2c and Cyp2j isoforms [12]). We focused on Cyp2c29, a major hepatic CYP monooxygenase, which accounts for approximately 70% of the total *Cyp2c/2j* mRNA content in mouse liver [13]. qRT-PCR showed that the gene expression of *CYP monooxygenases*, including *Cyp2c29*, *Cyp2c37*,

*Cyp2c38*, *Cyp2c39*, *Cyp2c40*, *Cyp2c50*, *Cyp2c55*, *Cyp2j5*, *Cyp2j6*, and *Cyp2j29*, was increased in the liver of SPF mice compared to GF mice (**Fig. 1E**). This result supports our hypothesis that gut microbiota elevates CYP eicosanoids in the liver by increasing the expression of CYP monooxygenases.

Previous studies have shown that some metabolites from the systemic circulation or the colon could enter the liver and subsequently alter the liver's metabolome [14]. Since CYP eicosanoids have a half-life in the circulation [15], we hypothesize that the gut microbiota's enhancing effects on CYP eicosanoids in the liver are not likely to be caused by the transfer of these eicosanoids from the circulation or the colon. To test this hypothesis, we analyzed CYP eicosanoids in the serum and colon of SPF mice versus GF mice. We found that the concentrations of CYP eicosanoids, including fatty acid epoxides and fatty acid diols, were not significantly changed in the colon and serum of SPF mice compared with those of GF mice (**Fig. S3-4**). This result supports the conclusion that eicosanoid transfer is unlikely to contribute to the microbiota's inducing effects on the liver's CYP eicosanoids. Together, our findings (**Fig. 1** and **Fig. S3-4**) support the conclusion that gut microbiota increases hepatic expression of CYP monooxygenases, elevating the concentrations of CYP eicosanoids in the liver.

To further test the roles of gut microbiota in modulating the liver's CYP eicosanoid pathway, we used another approach, the antibiotic-mediated suppression of gut microbiota (**Fig. S5A**). We employed a well-established antibiotic cocktail containing 1.0 g/L ampicillin and 0.5 g/L neomycin [16,17]. Our previous studies have shown that the cocktail effectively depletes gut microbiota in mice [18-21]. To further test this, we analyzed the total fecal microbial biomass using the *16S rRNA* gene as a marker [17]. Consistent with previous studies by us and others [16-21], the antibiotic treatment dramatically reduced the level of total fecal microbial biomass in mice (**Fig. S5B**), further supporting its microbiota-depleting effects. We found that compared with control

SPF C57BL/6 mice, the antibiotic-treated mice exhibited reduced expression of CYP monooxygenases, notably *Cyp2c29* (**Fig. S5C**) and lower levels of CYP eicosanoids (**Fig. S5D-E**) in the liver. These findings are consistent with the results observed in the GF mice (**Fig. 1**), further supporting the conclusion that gut microbiota upregulates the CYP eicosanoid pathway in the liver.

**Bacteria-derived indole induces the CYP eicosanoid pathway in the liver**

We investigated the mechanisms by which gut microbiota induces the liver's CYP eicosanoid pathway. Previous studies demonstrate that gut microbiota-produced small-molecule metabolites can translocate from the gut to the liver [14]. We hypothesize that the translocated metabolites induce the CYP eicosanoid pathway in the liver.

To test this hypothesis, we first examined the effects of a series of microbial metabolites on CYP monooxygenase expression in liver cells, including HepG2 liver cancer cells and HMCPMS primary liver cells (**Fig. 2A**). A recent study by Kampschulte et al. showed that CYP2C19 is a major enzyme involved in the biosynthesis of fatty acid epoxides [22], therefore we used CYP2C19 as a marker of CYP monooxygenases. Among the tested microbial metabolites, we found that indole, a microbial metabolite derived from tryptophan [23,24], showed the strongest effect on inducing *CYP2C19* expression in HepG2 cells (**Fig. 2B**). Indole derivatives, such as indole-3-aldehyde, indole-3-lactate, and indole-3-propionate, had no such effects (**Fig. 2B**). siRNA knockdown of pregnane X receptor (*PXR*), a nuclear receptor highly expressed in the liver that regulates CYP2C expression [25,26], abolished indole's inducing effects on *CYP2C19* expression, supporting that indole increases *CYP2C19* expression via PXR-dependent mechanisms (**Fig. 2C**). Besides HepG2 cells, indole treatment also induced *CYP2C19* expression in HMCPMS cells, a primary human liver cell line (**Fig. 2D**).

After demonstrating the effect of indole on CYP monooxygenase expression *in vitro*, we tested its effects *in vivo*. We treated SPF C57BL/6 mice with indole via drinking water (see the scheme of the experiment in **Fig. 2E**) and found that indole increased hepatic expression of CYP monooxygenases, including *Cyp2c29*, *Cyp2c37*, *Cyp2c38*, *Cyp2c44*, *Cyp2c50*, *Cyp2c54*, and *Cyp2c55* (**Fig. 2F**). Together, these results (**Fig. 2A-2F**) demonstrate that indole administration upregulates CYP monooxygenases *in vitro* and *in vivo*.

Having demonstrated that external treatment with indole upregulated CYP monooxygenases in liver cells and mice, we tested the effects of bacteria-derived indole. Previous studies have shown that the commensal *Bacteroides* are among the primary contributors to indole production in the gut microbiota [27]. We mono-colonized GF mice with either wild-type (WT) *B. thetaiotaomicron*, an indole-producing bacteria, or a mutant strain, *B. thetaiotaomicron* Δ1492, incapable of producing indole [27] (see tests of indole production by the WT and mutant strains in **Fig. S6** and the scheme of the germ-free mouse experiment in **Fig. 2G**). Compared with GF mice mono-colonized with *B. thetaiotaomicron* Δ1492, those mono-colonized with WT *B. thetaiotaomicron* had higher hepatic expression of CYP monooxygenases, including *Cyp2c29*, *Cyp2c37*, *Cyp2c44*, *Cyp2c50*, and *Cyp2c55* (**Fig. 2H**), and increased hepatic concentrations of CYP eicosanoids (**Fig. 2I**, see complete LC-MS/MS lipidomics data in **Table S4-S5**). Overall, these results support that gut microbes-derived indole induces the liver's CYP eicosanoid pathway.

**Bacteria/LPS translocation doesn't induce the liver's CYP eicosanoid pathway**

Previous studies demonstrate that under pathological conditions characterized by compromised gut barrier functions, gut bacteria or lipopolysaccharide (LPS) can move from the gut to the liver

[14], promoting the development of many liver diseases [28-31]. We tested whether bacteria/LPS translocation contributes to the microbiota's inducing effects on the liver's CYP eicosanoid pathway. Given that our experiments comparing SPF mice with GF or antibiotic-treated mice (**Fig. 1** and **Fig. S5**) were conducted on healthy mice with intact intestinal barriers and minimal bacteria/LPS translocation, we hypothesize that direct bacteria/LPS translocation is unlikely to make a major contribution to the observed effects.

To test this hypothesis, we first compared the levels of microbial species in the circulation of control SPF mice versus antibiotic-treated mice. We found that the levels of microbial species in the circulation of both control and antibiotic-treated mice were comparable to those in the negative control (PBS buffer), indicating a minimal presence of circulating bacterial species in both groups of mice. Moreover, there was little difference in circulating bacterial species between control mice with antibiotic-treated mice (**Fig. S7**). This result is consistent with previous findings [32], suggesting that direct bacteria/LPS translocation is unlikely to contribute to the observed microbiota's effects on the CYP eicosanoid pathway in the liver.

To further test this hypothesis, we compared the expression of CYP monooxygenases in WT mice versus mice deficient in Toll-like receptor 4 (TLR4), an important receptor that recognizes gut bacteria or LPS [33]. We didn't find significant differences in hepatic expression of CYP monooxygenases between WT mice with TLR4-knockout (KO) mice (**Fig. S8**). Overall, these results suggest that direct bacteria/LPS translocation is unlikely to play a significant role in the gut microbiota's effect on the induction of the CYP eicosanoid pathway in the liver.

**Gut microbiota protects the liver from chemically or surgically induced liver injury**

Previous studies have shown that GF animals develop more severe chemically or surgically induced liver injury and fibrosis compared to conventionally raised control animals, supporting the protective role of gut microbiota against liver injury and related disorders [3-10]. To further test this, we studied the extent to which antibiotic suppression of gut microbiota modulates the progression of liver injury in mice.

First, we used a well-established $CCl_4$-induced acute liver injury model to study the roles of gut microbiota in liver injury [34] (see the scheme of the experiment in **Fig. 3A**). We employed the antibiotic cocktail (1.0 g/L ampicillin and 0.5 g/L neomycin) that was used in previous studies [16-21] and our experiment above (**Fig. S5**). The antibiotic treatment further exacerbated $CCl_4$-induced liver injury, with increased plasma concentrations of aspartate transaminase (AST) and alanine aminotransferase (ALT), which are well-established markers of liver injury [35] (**Fig. 3B**). Additionally, H&E histology demonstrates that antibiotic treatment increased the severity of $CCl_4$-induced damage in the liver (**Fig. 3C**). Together, these results showed that antibiotic suppression of gut microbiota exacerbates $CCl_4$-induced liver injury, illustrating the gut microbiota's hepatoprotective effects, akin to previous studies using GF animal models [3-10].

To further test the roles of gut microbiota in liver injury, we used another disease model, the partial hepatectomy surgery-induced liver injury [36] (see the scheme of the experiment in **Fig. 3D**). Consistent with the results above (**Fig. 3A-C**), we found that antibiotic suppression of gut microbiota increased the development of hepatectomy-induced liver injury, as assessed by plasma levels of AST and ALT and histology analysis of the liver (**Fig. 3E-F**). Together, these results (**Fig. 3**) support that the gut microbiota protects the liver from both chemically and surgically induced liver injuries, consistent with previous studies [3-10].

**Pharmacological inhibition or genetic ablation of CYP monooxygenases exacerbates chemically induced liver injury**

The mechanisms by which gut microbiota protects the liver from injury are not well understood. Having demonstrated that gut microbiota induces the liver's CYP eicosanoid pathway (**Fig.1** and **Fig S5**), we hypothesize that the microbiota-liver-CYP axis contributes to the gut microbiota's hepatoprotective effects. To test this hypothesis, we examined the role of CYP monooxygenases in the development of liver injury.

First, we studied whether the expression of CYP monooxygenases is altered in $CCl_4$-induced liver injury. Compared with control mice, the expression of a series of CYP monooxygenases, including *Cyp2c29*, *Cyp2c37*, *Cyp2c38*, *Cyp2c40*, *Cyp2c44*, *Cyp2c50*, *Cyp2c54*, *Cyp2j5*, *Cyp2j8*, and *Cyp2j9*, is reduced in the liver of $CCl_4$-treated mice (**Fig. S9**). These results suggest that the CYP monooxygenases are downregulated in liver injury.

Next, we determined the extent to which inhibition of CYP monooxygenases modulates the development of $CCl_4$-induced liver injury. To do so, we orally gavaged the mice with two different CYP monooxygenase inhibitors, SKF-525A and clotrimazole [37], then stimulated the mice with $CCl_4$ to induce liver injury (see the scheme of the experiment in **Fig. S10A**). Administration of the CYP inhibitor increased the severity of $CCl_4$-induced liver injury, as assessed by plasma levels of ALT and AST, and histology analysis of the liver (**Fig. S10B-C**). These results are consistent with our hypothesis, supporting that the CYP monooxygenases have a protective effect on $CCl_4$-induced liver injury.

Finally, to test the role of CYP monooxygenases in liver injury, we employed genetically engineered mice deficient in CYP monooxygenases. Graves et al. showed that *Cyp2c29* is the most abundant Cyp2c/2j monooxygenase in mouse liver: *Cyp2c29* makes up about 70% of total *Cyp2c/2j* mRNA content in mouse liver [13]. Therefore, we generated *Cyp2c29* KO mice to study the role of CYP monooxygenases in $CCl_4$-induced liver injury (see the scheme of the experiment in **Fig. 4A**). The expression of *Cyp2e1*, a major enzyme involved in the metabolic activation of $CCl_4$ [38], was not changed in *Cyp2c29* KO mice compared with WT mice (**Fig. S11**), supporting that it is feasible to perform the $CCl_4$ model in Cyp2c29 KO mice. Compared with $CCl_4$-stimulated WT littermates, the $CCl_4$-stimulated *Cyp2c29* KO mice had elevated levels of AST and ALT in the plasma (**Fig. 4B**), higher gene expression of pro-inflammatory cytokines (*Tnf-α*, *Il-1β*, *Il-6*, and *Ccl2*) in the liver (**Fig. 4C**), and more severe liver damage (**Fig. 4D**). This result demonstrates that genetic ablation of *Cyp2c29* exacerbates $CCl_4$-induced liver injury. LC-MS/MS showed that the concentrations of CYP eicosanoids were reduced in the livers of *Cyp2c29* KO mice compared with those of the WT mice (**Fig. 4E**), supporting the target engagement of *Cyp2c29* deletion on the CYP eicosanoid pathway. Overall, these results are consistent with our findings above using the CYP inhibitors (**Fig. S10**), supporting the conclusion that the CYP eicosanoid pathway protects the liver from chemically induced liver injury.

**Pharmacological inhibition or genetic ablation of CYP monooxygenases exacerbates surgically induced liver injury**

To further test the roles of CYP monooxygenases in liver injury, we used another model, the hepatectomy surgery-induced liver injury [36]. We tested the extent to which inhibition or genetic ablation of CYP monooxygenases modulates the development of hepatectomy-induced liver injury.

First, we treated mice with the CYP monooxygenase inhibitor SKF-525A or clotrimazole [37], then performed hepatectomy surgery to induce liver injury (see the scheme of the experiment in **Fig. S12A**). Compared with vehicle-treated mice, the mice treated with the CYP inhibitors, notably clotrimazole, displayed higher concentrations of plasma ALT and AST, and more severe liver damage (**Fig. S12B-C**). Next, we compared the development of hepatectomy-induced liver injury in WT mice versus *Cyp2c29* KO mice (see the scheme of the experiment in **Fig. 4F**). Compared with WT mice, the *Cyp2c29* KO mice displayed more severe liver damage, as assessed by circulating concentrations of ALT and AST, and H&E histology evaluation of the liver tissue (**Fig. 4G-H**). Together, these results (**Fig. S12** and **Fig. 4**) support that inhibition or genetic ablation of CYP monooxygenases exacerbates surgically induced liver injury, further supporting the protective effects of CYP monooxygenases on liver injury.

**Genetic ablation of CYP monooxygenases exacerbates chemically induced liver fibrosis**

Persistent liver injury could lead to liver fibrosis [2]. We used *Cyp2c29* KO mice to study the roles of the CYP monooxygenase pathway in chronic $CCl_4$ treatment-induced liver fibrosis (see the scheme of the experiment in **Fig. 5A**). Compared with $CCl_4$-stimulated WT mice, the $CCl_4$-stimulated *Cyp2c29* KO mice exhibited higher circulating levels of ALT and AST (**Fig. 5B**), demonstrating that the *Cyp2c29* KO mice developed more severe liver injury. qRT-PCR analysis showed that *Cyp2c29* KO mice exhibited increased hepatic expressions of fibrosis markers including *Timp*, *Ctgf*, and *Collagen 1* compared with WT mice (**Fig. 5C**). Additionally, H&E and Masson's staining showed that compared with WT mice, the *Cyp2c29* KO mice have more severe liver damage and fibrosis (**Fig. 5D).** Overall, these results support the conclusion that the CYP monooxygenase pathway protects the liver from chronic $CCl_4$ treatment-induced liver fibrosis.

**Induction of CYP monooxygenases by indole ameliorates liver injury and fibrosis**

Having shown that experimental disruption of the microbiota-liver-CYP axis worsened liver injury and fibrosis, we tested the extent to which indole administration, which induces the liver's CYP eicosanoid pathway (**Fig. 2**), attenuates the progression of liver injury or fibrosis in mice.

We treated mice with drinking water with or without indole, then stimulated the mice with $CCl_4$ to induce acute liver injury (see the scheme of the experiment in **Fig. 6A**). Compared with vehicle-treated $CCl_4$-stimulated mice, the indole-treated $CCl_4$ mice had reduced levels of ALT and AST (**Fig. 6B**). H&E histology showed that indole treatment attenuated $CCl_4$-induced liver damage (**Fig. 6C**). Furthermore, qRT-PCR showed that indole increased the expression of CYP monooxygenases, including *Cyp2c29*, *Cyp2c38*, *Cyp2c44*, *Cyp2c50*, and *Cyp2c54*, in the liver (**Fig. 6D**). These results demonstrate the liver protective effects of indole, further supporting the conclusion that the microbiota-liver-CYP axis protects the liver against liver injury.

We next evaluated the effect of indole on liver fibrosis induced by chronic $CCl_4$ treatment (see the experimental scheme in **Fig. 6E**). Indole administration reduced plasma levels of ALT and AST, decreased the gene expression of fibrosis markers such as *Timp*, *Ctgf*, and *Collagen 1* in the liver, and mitigated the severity of liver damage and fibrosis (**Fig. 6F-6H**). Additionally, indole treatment increased the hepatic expression of *Cyp2c* monooxygenases, notably *Cyp2c29* (**Fig. 6I**). Collectively, these results demonstrate that systemic administration of indole induces the CYP monooxygenase pathway and protects against liver fibrosis, further highlighting the critical role of the microbiota-liver-CYP axis in liver disorders.

## Discussion

Substantial studies support that the presence of gut microbiota protects the liver against liver injury and fibrosis, but the specific gut microbial factors or pathways involved remain poorly defined [4-10]. Here, our central finding is that gut microbiota-derived small-molecule metabolites, notably indole, upregulate the CYP eicosanoid pathway in the liver, protecting the liver against liver injury and fibrosis (see the proposed model in **Fig. S13**). This finding provides a unique molecular mechanism by which gut microbiota interacts with the liver, modulating host metabolism and disease development. In addition, the gut-liver-CYP axis could be therapeutically explored to ameliorate these liver diseases.

Previous research about the biosynthesis and function of eicosanoids has focused on the host factors, while the role of gut microbiota involved in these processes is unknown [11,12]. Here, we demonstrate that gut microbiota induces the expression of Cyp2c monooxygenases and elevates the concentrations of CYP eicosanoids in the liver. These findings are consistent with previous research demonstrating the gut microbiota's role in enhancing *Cyp2c* expression in the liver, though previous studies only focused on gene expression and did not measure the concentrations of CYP-derived eicosanoids or other metabolites [39-42]. Nevertheless, there are conflicting results from previous studies, with some showing that GF mice exhibit increased or unaltered expression of *Cyp2c* genes in the liver compared to conventional mice [43-46]. These inconsistencies may, in part, be attributed to differences in experimental conditions when comparing GF and conventional mice. For instance, the gut microbiota composition of conventional mice can vary across different animal facilities. In our experiment, we strictly controlled animal experiment procedures, including providing both GF and control SPF mice with the same autoclaved mouse diet and drinking water. Additionally, we employed other strategies such as using antibiotic-treated mice and mono-colonizing GF mice with wild-type or mutant *B. thetaiotaomicron* to investigate the impact of gut bacteria on the liver's CYP eicosanoid pathway. Consistently, we observed that gut microbiota upregulates the liver's CYP eicosanoid pathway. Together, these studies support that gut microbiota plays an important role in regulating host eicosanoid metabolism.

Using a combination of approaches, including external treatment with indole in liver cells (both HepG2 liver cancer cells and HMCPMS primary liver cells) and in SPF mice, as well as mono-colonizing GF mice with WT or mutant indole-producing *B. thetaiotaomicron*, our findings support the critical role of indole in mediating the microbiota's inducing effect on the CYP eicosanoid pathway in the liver. siRNA-mediated knockdown of PXR, a nuclear receptor highly expressed in the liver and a regulator of Cyp2c expression [25,26], abolished the Cyp2c-inducing effects of indole *in vitro*. This result supports that indole augments CYP monooxygenase expression through PXR-dependent mechanisms, aligning with recent research demonstrating indole as a PXR ligand [47]. Together, our findings support a model that gut microbiota-derived indole moves from the gut to the liver, likely via the portal vein, interacting with the PXR receptor and inducing the CYP eicosanoid pathway in the liver. Beyond indole, it is plausible that other gut microbial metabolites, particularly the metabolites that are PXR ligands, may also induce the CYP eicosanoid pathway in the liver, conferring hepatoprotective effects. Identification of these beneficial metabolites will enhance our understanding of microbiota-liver interactions and pave the way for developing microbiota-based strategies for reducing the risks of liver diseases.

Many liver diseases are characterized by intestinal barrier dysfunctions, which lead to increased translocation of LPS from the gut into the liver, promoting the progression of many liver diseases, including liver cancer [28], nonalcoholic fatty liver disease [29,30], and alcoholic liver disease [31]. Here, we investigated whether LPS translocation contributes to the observed effects of the microbiota. Our data, however, suggest that LPS translocation is unlikely to contribute to the observed microbiota's inducing effects on the CYP eicosanoid pathway in the liver. This conclusion is consistent with previous findings. Firstly, previous studies have shown that external administration of LPS reduces the expression of *Cyp2c/2j* in the liver [13,48]. Secondly, genetically engineered mice deficient in the LPS-TLR4 pathway have exhibited attenuated development of liver injury and fibrosis, indicating that LPS promotes the progression of these liver disorders [49-51]. These findings implicate that, opposite the effects of indole, LPS reduces the expression of the liver's CYP monooxygenases and promotes the development of liver injury and fibrosis. The contrasting effects of LPS and indole suggest the importance of the gut-liver-CYP axis in the pathogenesis of liver injury and fibrosis. Under a healthy gut condition, gut microbiota-derived small-molecular metabolites, such as indole, translocate to the liver via portal vein, inducing the liver's CYP eicosanoid pathway and protecting against liver damage and fibrosis. In contrast, when the gut barrier functions are disrupted, LPS or gut microbes can move to the liver, suppressing the hepatic CYP pathway and promoting the progression of these disorders.

Our results support that the CYP eicosanoid pathway plays an important role in the progression of liver injury and fibrosis, protecting the liver from these disorders. Firstly, we observed a reduction in the expression of *Cyp2c/2j* isoforms in $CCl_4$-induced liver injury, and this finding is consistent with a recent study demonstrating reduced hepatic expression of *Cyp2c* in liver cirrhosis induced by $CCl_4$ or bile duct ligation [52]. Human studies also indicate the downregulation of the CYP eicosanoid pathway in liver fibrosis and related conditions. Arvind et al. employed LC-MS/MS to assess circulating concentrations of CYP-derived eicosanoids in nonalcoholic fatty liver disease (NAFLD) patients and identified a negative association between CYP monooxygenase function and fibrosis severity [53]. Collectively, these findings support the notion that the CYP monooxygenase pathway is downregulated in liver injury and fibrosis. Secondly, we demonstrated that inhibition of the CYP eicosanoid pathway, achieved through antibiotic treatment, pharmacological CYP inhibitors, or genetically engineered mice deficient in *Cyp2c29* monooxygenase, increased the severity of chemically or surgically liver injury or fibrosis. In contrast, induction of the CYP pathway via indole treatment conferred protection against liver injury and fibrosis. In summary, these results support that the CYP eicosanoid pathway safeguards the liver against injury and fibrosis and contributes to the mechanisms underlying the hepatoprotective effects of gut microbiota. These findings could have significant implications for human translation, as pharmacological inhibitors of soluble epoxide hydrolase (sEH), the major enzyme that degrades CYP-derived eicosanoids, are being evaluated in human clinical trials for other human disorders [54].

In summary, our central finding is that specific gut microbial metabolites, notably indole, translocate from the gut to the liver, activating the CYP eicosanoid pathway and protecting the liver against liver injury and fibrosis. These findings enhance our understanding of the mechanisms underlying gut-microbiota interactions. Moreover, the gut-liver-CYP axis presents a promising therapeutic target for developing innovative strategies to treat or prevent liver injury, fibrosis, and related diseases.

## Materials and Methods

### Study design

The study aims to test the hypothesis that gut microbiota modulates the biosynthesis of tissue-protective eicosanoids in the liver, which subsequently protects the liver from injury and fibrosis. First, we used LC-MS/MS-based lipidomics to compare eicosanoid profiles in the livers of conventionally raised mice with those of GF or antibiotic-treated mice. This approach revealed that gut microbiota induced the CYP eicosanoid pathway in the liver. Next, we investigated the mechanisms by which gut microbiota induced the CYP eicosanoid pathway in the liver, employing a combination of strategies including bacterial metabolite screening, external treatment with the identified bacterial metabolite in liver cells (HepG2 and HMCPMS) and in mice, and mono-colonization of GF mice with WT or mutant gut bacteria. Finally, we assessed the functional roles of the CYP eicosanoid pathway in the development of chemically or surgically-induced liver injury and fibrosis. For each animal experiment, the mice were randomized to receive treatment. The number of mice and the method of statistical analysis are described in the Figure Legends or Materials and Methods.

**Animal experiments**

The animal experiments were conducted following protocols approved by the Institutional Animal Care and Use Committees of the University of Massachusetts-Amherst, National University of Singapore, Jiangsu University, China Agricultural University, and Massachusetts Host-Microbiome Center at the Brigham and Women's Hospital (BWH).

**Animal experiment 1: Analysis of the CYP eicosanoid pathway in the liver of SPF mice *vs.* GF mice**

The SPF and GF mouse experiment was performed at the Massachusetts Host-Microbiome Center at BWH. SPF C57BL/6 mice (age = 4 weeks) were obtained from Taconic Biosciences. After arrival, the mice were housed in a standard SPF mouse facility at the Massachusetts Host-Microbiome Center. They were provided with autoclaved tap water and maintained on autoclaved autoclavable LabDiet 5021 (LabDiet) for 2 weeks. At the age of 6 weeks, the mice were euthanized, and tissues were collected for analysis. GF C57BL/6 mice were obtained from the Massachusetts Host-Microbiome Center. These mice were housed in GF isolators, provided with the same autoclaved tap water, and received the same autoclaved LabDiet 5021. At the age of 6 weeks, the GF mice were euthanized for tissue collection and analysis.

**Animal experiment 2: Analysis of the CYP eicosanoid pathway in the liver of control mice *vs.* antibiotic-treated mice**

SPF C57BL/6 mice (age = 6 weeks) were treated with drinking water with or without an antibiotic cocktail (1.0 g/L ampicillin and 0.5 g/L neomycin) for 1 week [16-21]. At the end of the experiment, the mice were euthanized to collect tissues for analysis. To check the effect of the antibiotic treatment on suppressing gut microbiota, mouse fecal samples were collected to analyze the total fecal microbial biomass using the *16S rRNA* gene as a marker [17].

**Animal experiment 3: Effects of indole administration on liver's CYP eicosanoid pathway in mice**

SPF C57BL/6 mice (age = 6 weeks) were treated with or without 10 μM indole dissolved in drinking water. After 1 week, the mice were euthanized for analysis.

**Animal experiment 4: Mono-colonization GF mice with WT *B. thetaiotaomicron* or *B. thetaiotaomicron* Δ1492**

WT *B. thetaiotaomicron* and *B. thetaiotaomicron* Δ1492 were kindly provided by Dr. Michael Fischbach at Stanford University [27]. Indole production by the bacteria was tested using Ehrlich's reagent [27]. The GF mouse experiment was performed at the Massachusetts Host-Microbiome Center at BWH. Pure isolates of WT *B. thetaiotaomicron* or *B. thetaiotaomicron* Δ1492 were cultured overnight in the brain heart infusion medium. Then, 0.2 mL of the overnight culture (5.5-7.5 × $10^8$ CFU/mL) per mouse was orally gavaged to 6-week-old C57BL/6J GF mice. One week post-inoculation, fecal samples were collected to confirm stable colonization of the gavaged bacteria in the gut. Three weeks post-inoculation, the mice were euthanized for analysis.

**Animal experiment 5: Analysis of gene expression of *Cyp2c/2j monooxygenase* in WT mice *vs. Tlr4*$^{-/-}$ mice**

WT mice and *Tlr4*$^{-/-}$ mice, established on C57BL/6 background, were obtained from the Jackson laboratory. The mice were housed in a standard SPF animal facility and fed with standard chow for 4-6 weeks, then the mice were euthanized to collect tissues for analysis.

**Animal experiment 6: Effects of antibiotic suppression of gut microbiota on $CCl_4$-induced liver injury in mice**

SPF C57BL/6 mice (age = 6 weeks) were treated with drinking water with or without the antibiotic cocktail (1.0 g/L ampicillin and 0.5 g/L neomycin) throughout the whole period of the experiment [16-21]. After 1 week, the mice were treated with a one-time i.p. injection of $CCl_4$ (dose = 0.5 µL/g body weight) dissolved in vegetable oil [34]. After another 2 days, the mice were euthanized for analysis. The plasma concentrations of AST and ALT, which are well-established markers of liver injury [35], were measured using an ELISA kit (Thermo Fisher Scientific).

**Animal experiment 7: Effects of antibiotic suppression of gut microbiota on partial liver hepatectomy-induced liver injury in mice**

SPF C57BL/6 mice (age = 6 weeks) were treated with drinking water with or without the antibiotic cocktail (1.0 g/L ampicillin and 0.5 g/L neomycin) throughout the whole period of the experiment. Following a 1-week treatment period, partial hepatectomy surgery was conducted, involving the removal of approximately two-thirds of the liver. After an additional 2 days, the mice were euthanized for analysis.

**Animal experiment 8: Effects of CYP monooxygenase inhibitors on $CCl_4$- or hepatectomy-induced liver injury**

SPF C57BL/6 mice (age = 6 weeks) were treated with two different CYP inhibitors, clotrimazole or SKF525 (dose = 20 mg/kg), dissolved in polyethylene glycol 400 by oral gavage, once per day, throughout the whole period of the experiment. After 7 days, the mice were treated with a one-time i.p. injection of $CCl_4$ (dose = 0.5 μL/g body weight) dissolved in vegetable oil [34], or treated with hepatectomy surgery, as described above. After another 2 days, the mice were euthanized for analysis.

**Animal experiment 9: $CCl_4$- or hepatectomy-induced liver injury in WT mice *vs. Cyp2c29*$^{-/-}$ mice**

*Cyp2c29*$^{-/-}$ mice and WT littermates (age = 6 weeks), established on the C57BL/6 background, were obtained from Gempharmatech (Nanjing, China). The mice were treated with a one-time i.p. injection of $CCl_4$ (dose = 0.5 μL/g body weight) dissolved in vegetable oil [34], or treated with hepatectomy surgery, to induce liver injury. After another 2 days, the mice were euthanized for analysis. The concentrations of free-form CYP eicosanoids in the liver tissues were analyzed by LC-MS/MS (LipidALL Technologies, Changzhou, China).

**Animal experiment 10: Chronic $CCl_4$-induced chronic fibrosis in on WT mice *vs. Cyp2c29*$^{-/-}$ mice**

*Cyp2c29*$^{-/-}$ mice and WT littermates (age = 6 weeks) were treated with $CCl_4$ (dose = 0.2 μL/g body weight) by i.p. injection, 3 times per week, for a total of 4 weeks. At the end of the experiment, the mice were euthanized for analysis.

**Animal experiment 11: Effects of indole on $CCl_4$-induced liver injury or fibrosis**

C57BL/6 mice (age = 6 weeks) were treated with or without 10 μM indole dissolved in drinking water throughout the whole period of the experiment. Then the mice were treated with $CCl_4$ to induce liver injury or fibrosis, as described above. At the end of the experiment, the mice were euthanized for analysis.

### Histology

Liver tissues were fixed in 4% paraformaldehyde for 24 h and embedded in paraffin. The tissues are sliced into 5-um sections and dewaxed in serial xylene and rehydrated through graded ethanol. Then, the sections were stained with hematoxylin and eosin. After sealing the sections with neutral gum, they were analyzed for inflammation and tissue damage using a light microscope (Nikon). For the assessment of liver fibrosis, Masson's trichrome staining was performed. The tissue slices were immersed in a Masson dye solution set (Servicebio), then washed with 1% glacial acetic acid, dehydrated with anhydrous ethanol, and finally sealed with neutral gum. The tissues were then observed under an upright optical microscope (Nikon). The H&E and Masson’s staining images were analyzed by image J. The ratio of the necrotic area and fibrosis area was calculated by the positive area divided by the total area in H&E and Masson’s staining images, respectively.

### Quantitative real-time reverse-transcription PCR (qRT-PCR)

Total RNA was isolated from the liver tissues using TRIzol (Thermo Fisher Scientific) following the manufacturer’s instructions. The RNA was reverse transcribed into cDNA using a GoScript Reverse Transcriptase kit (Promega). qRT-PCR was carried out with a StepOne Real-Time PCR System (Thermo Fisher Scientific) with GoTaq master mixes (Promega). The sequences of the primers used in qRT-PCR are listed in **Table S6**. Glyceraldehyde-3-phosphate dehydrogenase (*Gapdh*) was used as an internal control.

### LC-MS/MS lipidomics analysis of eicosanoids in the liver of Ctrl mice vs. GF or antibiotic-treated mice

Liver samples were weighed and placed in tubes containing 9 µL of HBSS/mg liver. Subsequently, each tube was supplemented with 1 µL of 1 mM TPPU in methanol. Liver tissue was homogenized using a Tissuelyzer II (Qiagen) set to 30 Hz for 10 minutes. To extract free eicosanoids from the liver tissue, 10 µL internal standard mix was added to 100 µL (10 mg of liver homogenate) and extracted by liquid: liquid extraction with 600 µL ethyl acetate and dried under vacuum centrifugation. For the extraction of "total" eicosanoids, encompassing both free eicosanoids and those esterified at the sn-2 position of membrane phospholipids, a separate 100 µL aliquot (representing 10 mg of lysate) was incubated with 3 units of porcine pancreas PLA2 (Sigma-Aldrich). This step facilitated the hydrolysis of sn-2-esterified eicosanoids before undergoing identical liquid-liquid extraction. The extracted eicosanoids underwent analysis using LC-MS/MS, conducted on an Ultimate 3000 UHPLC equipped with an Xselect CSH C18, 2.1 × 50 mm, 3.5 µm particle column (Waters) and a TSQ Quantiva tandem mass spectrometer (Thermo Fisher Scientific). Quantification was established through multiple reaction monitoring and executed by a blinded observer employing TraceFinder (v4.1, ThermoFisher Scientific). Relative response ratios of analytes were compared to standard curves generated with eicosanoid standards from Cayman Chemicals.

**Effects of gut microbial metabolites on gene expression of *CYP monooxygenase* in HepG2 cells**

HepG2 liver cancer cells (ATCC) were cultured in Dulbecco’s Modified Eagle Medium (DMEM) (Gibco) fortified with 10 % fetal bovine serum (Gibco), 100 unit/mL penicillin, and 100 mg/mL streptomycin (Gibco) at 37 °C under an atmosphere with 5% $CO_2$. The cells were treated with a series of gut microbial metabolites, including indole-3-carboxaldehyde, indole-3-lactate, indole-3-propionate, lithocholic acid, sodium deoxycholate, lactate, kynurenine, 5-HT, and indole, or the vehicle (DMSO), for 24 h. The concentration of the test compound was 10 µM. At the end of the experiment, gene expression of *CYP2C19* was analyzed by qRT-PCR.

**Effects of indole on gene expression of *CYP2C19* in primary human liver cells**

Primary human liver cells (HMCPMS cells from Thermo Fisher Scientific) were cultured in DMEM medium fortified with 10 % fetal bovine serum (Gibco), 100 unit/mL penicillin, and 100 mg/mL streptomycin (Gibco) at 37 °C under an atmosphere with 5% $CO_2$. The cells were treated with indole, or the vehicle (DMSO), for 24 h. The concentration of the test compound was 10 μM. At the end of the experiment, gene expression of *CYP2C19* was analyzed by qRT-PCR.

**siRNA transfection**

siRNA duplexes of PXR oligos were purchased from Silencer™ Select siRNAs (Thermo Fisher Scientific). The siRNA oligos were transfected into cells using Lipofectamine RNAiMAX transfection reagent (Thermo Fisher Scientific) according to the manufacturer's instructions. The Silencer™ Select Negative Control was used as the negative control.

**Measurement of the abundance of TLR4 ligands using a TLR4 reporter assay**

HEK-Blue mTLR4 cells (InvivoGen) were cultured in DMEM medium containing 10% FBS and HEK-Blue Selection (InvivoGen) at 37 °C under an atmosphere with 5% $CO_2$, following the manufacturer's instructions. To measure the abundance of TLR4 ligands (e.g., LPS and bacteria) in mouse plasma, 10 μL mouse plasma sample and 190 μL of $2 \times 10^4$ HEK-Blue mTLR4 cells suspended in HEK-Blue detection medium, were added into each well of a 96-well plate. After 12 h incubation in a 37 °C incubator, the production of secreted embryonic alkaline phosphatase was determined by measuring the absorbance at 620 nm with a plate reader (Molecular Devices).

**Statical Analysis**

Data are expressed as mean ± standard error of the mean (SEM). Statistical comparison of two groups was performed using Student's t-test and comparison of more than two groups was performed using one-way ANOVA. $P < 0.05$ was considered statistically significant. The statistical analyses were performed using GraphPad Prism 8.0 statistical software (GraphPad Software).

**Acknowledgments**

We thank Dr. Michael Fischbach at Stanford University for kindly providing the gifts of WT and mutant *B. thetaiotaomicron* bacteria. We thank Drs. Bruce Hammock, David Mills, Fawaz Haj, and Ming-Fo Hsu at the University of California Davis, and Dr. Haojie Yu at National University of Singapore for discussion and help. This research is supported by new faculty start-up grants from the National University of Singapore and the University of California Davis (to G.Z.), a new faculty start-up grant from China Agricultural University (to H.Y.), and the Intramural Research Program of the NIH, National Institute of Environmental Health Sciences Z01 ES025034 (to D.C.Z). We acknowledge the NIH grant P30DK034854 and the use of the Harvard Digestive Disease Center's (HDDC's) core services, resources, technology, and expertise.

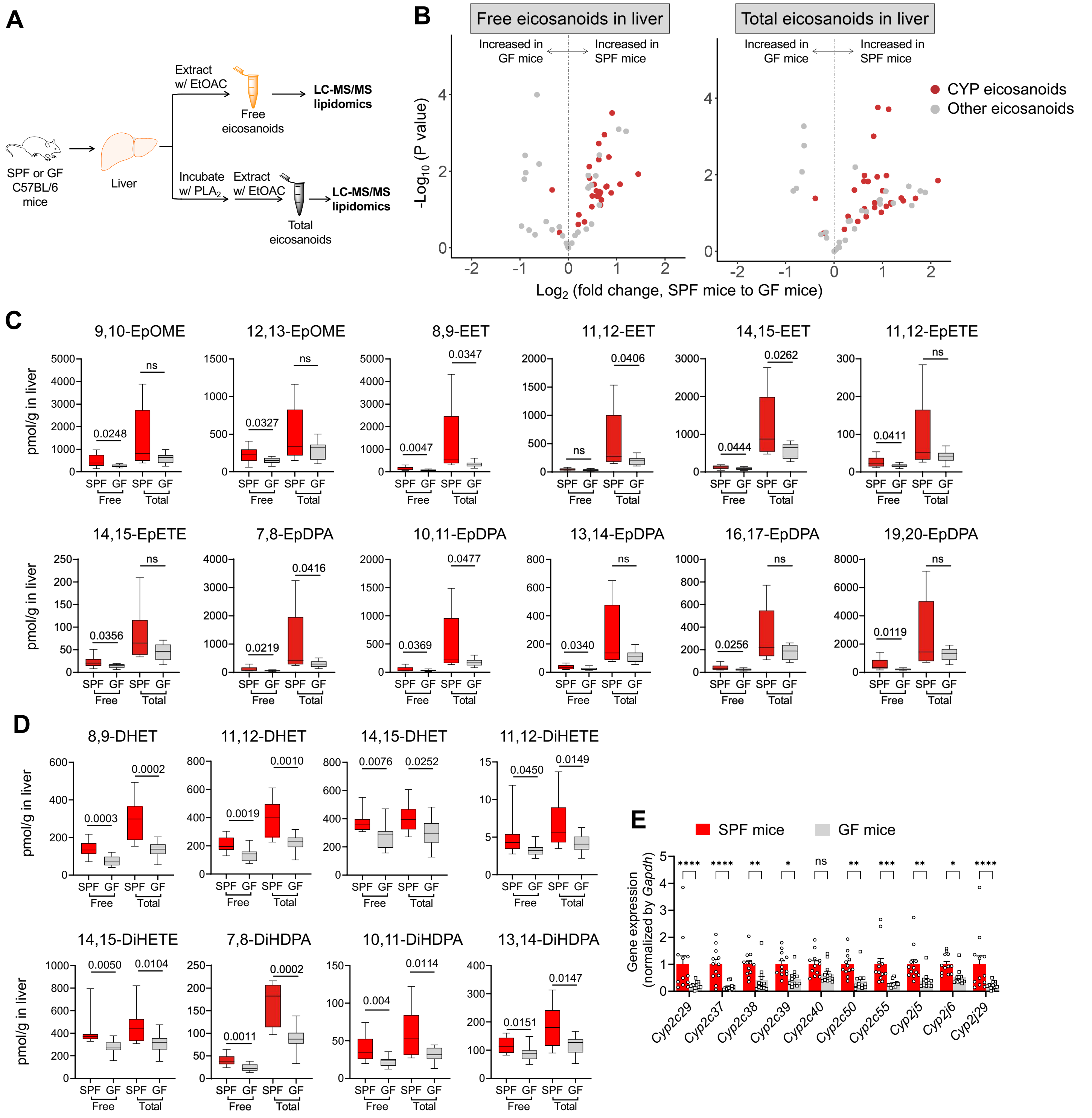

**Fig. 1. Gut microbiota induces the CYP eicosanoid pathway in the liver. A.** We used LC-MS/MS lipidomics to measure free and total eicosanoids in the liver of specific pathogen-free (SPF) and germ-free (GF) C57BL/6 mice (see experimental details in Materials and Methods). **B.** Volcano plot of the metabolites detected in the liver of SPF and GF mice. Each plotted point represents an eicosanoid metabolite. The red points represent fatty acid epoxides and/or diols which are produced by the CYP pathway, and the grey points represent the eicosanoids that are produced by other pathways such as COX and LOX. The X-axis shows the $\log_2$ of the fold of change of the eicosanoid detected in the liver of SPF mice *vs.* that of the GF mice. The Y-axis shows the $-\log_{10}$ of the *P* value calculated by using Welch's t-test. **C.** CYP-produced fatty acid epoxides are increased in the livers of SPF mice compared to those of GF mice. Free: free eicosanoids, Total: total eicosanoids. **D.** Fatty acid diols, which are downstream metabolites of fatty acid epoxides, are also increased in the livers of SPF mice compared to those of GF mice. **E.** Gene expression of CYP monooxygenases, notably *Cyp2c29*, the most abundant *Cyp2c/2j* monooxygenase in mouse liver, is increased in the livers of SPF mice compared to those of GF mice. The data are mean ± SEM, n = 11-12 mice per group. * $P<0.05$, ** $P<0.01$, *** $P<0.001$, and **** $P<0.0001$.

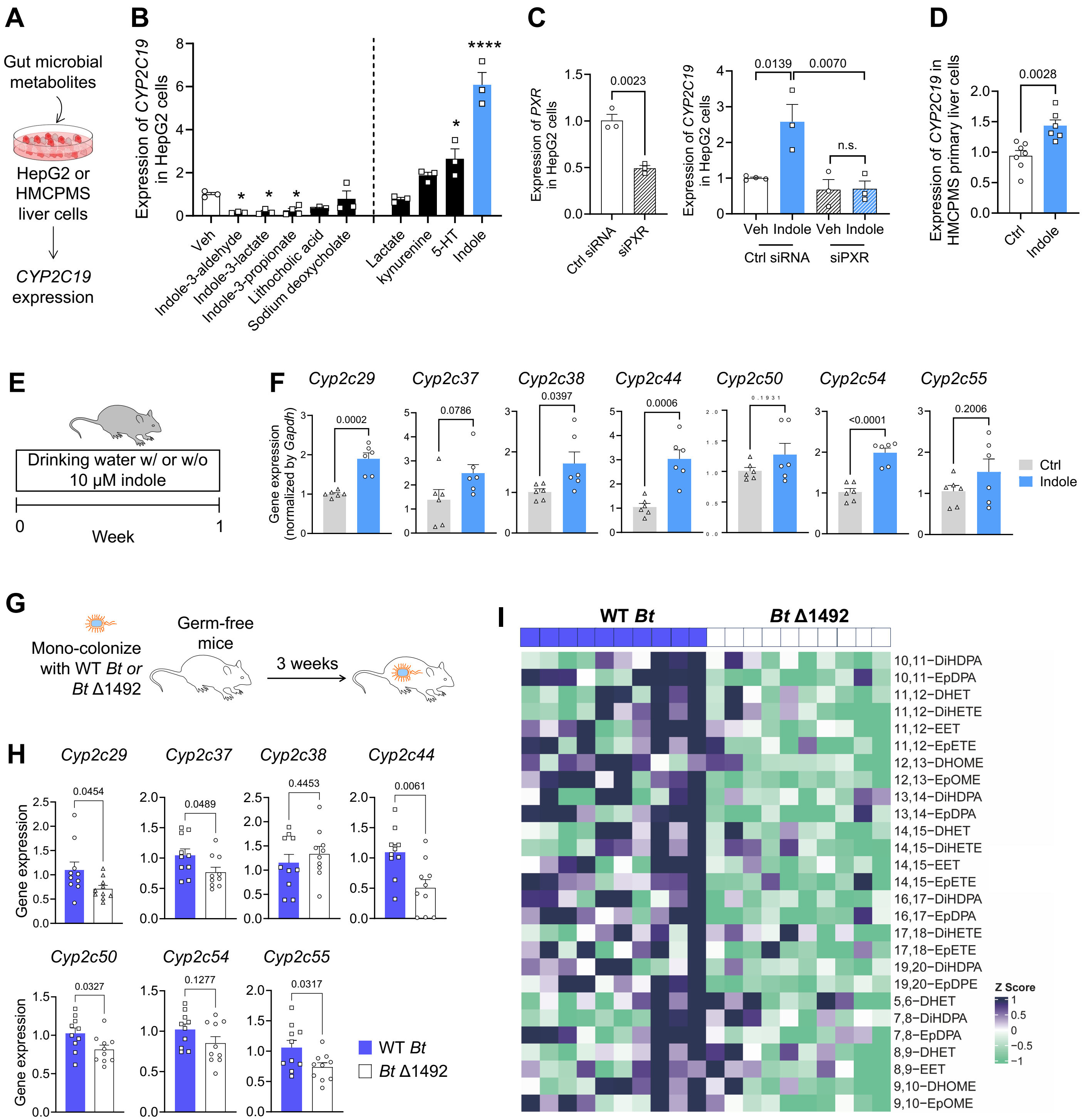

**Fig. 2. Gut bacteria-derived indole induces the liver's CYP eicosanoid pathway. A.** We tested the effects of a series of gut microbiota-produced small-molecule metabolites on *CYP2C19* expression in HepG2 or HMCPMS liver cells. **B.** Among the tested microbial metabolites, indole shows the most potent effect to induce gene expression of *CYP2C19* in HepG2 cells. **C.** siRNA knockdown of *Pregnane X receptor* (*PXR*) abolishes the *CYP2C19*-inducing effect of indole in HepG2 cells. **D.** Indole increased *CYP2C19* expression in HMCPMS cells, a primary human liver cell line. **E.** We treated SPF C57BL/6 mice with drinking water with or without 10 μM indole for 1 week. **F.** Indole treatment increases gene expression of *Cyp2c* in mouse liver (n = 6 mice per group). **G.** We mono-colonized germ-free C57BL/6 mice with wild-type (WT) *Bacteroides thetaiotaomicron* (*Bt*), an indole-producing bacteria, or a mutant strain, *Bt* Δ1492, incapable of producing indole. **H.** Compared to the germ-free mice colonized with *Bt* Δ1492, those colonized with WT *Bt* have higher expression of *Cyp2c* in the liver (n = 10 mice per group). **I.** Compared to the germ-free mice colonized with *Bt* Δ1492, those colonized with WT *Bt* have increased concentrations of CYP eicosanoids (total eicosanoids, a combination of free CYP eicosanoids and membrane phospholipid-incorporated CYP eicosanoid esters) in the liver (n = 10 mice per group). The data are mean ± SEM, * $P<0.05$, **** $P<0.0001$.

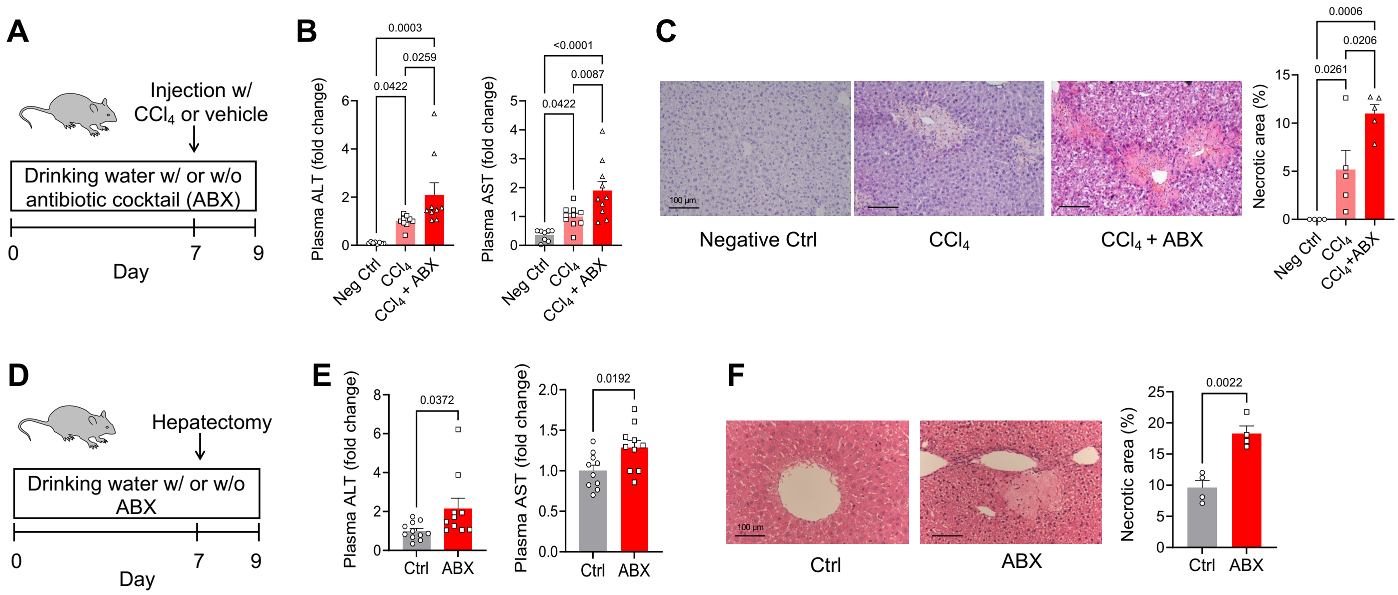

**Fig. 3. Antibiotic suppression of gut microbiota exacerbates chemically and surgically induced liver injury in mice. A.** We treated C57BL/6 mice with drinking water with or without an antibiotic cocktail (1.0 g/L ampicillin and 0.5 g/L neomycin) and then stimulated the mice with $CCl_4$ to induce liver injury. **B.** Antibiotic treatment increases plasma concentrations of AST and ALT in mice (n = 8-10 mice per group). **C.** H&E histology shows that antibiotic treatment exacerbates liver damage (n = 4-5 mice per group). **D.** We treated C57BL/6 mice with drinking water with or without an antibiotic cocktail and then induced liver injury via hepatectomy surgery. **E.** Antibiotic treatment increases plasma concentrations of AST and ALT in mice (n = 10-11 mice per group). **F.** H&E histology shows that antibiotic treatment exacerbates liver damage (n = 4 mice per group). The data are mean ± SEM.

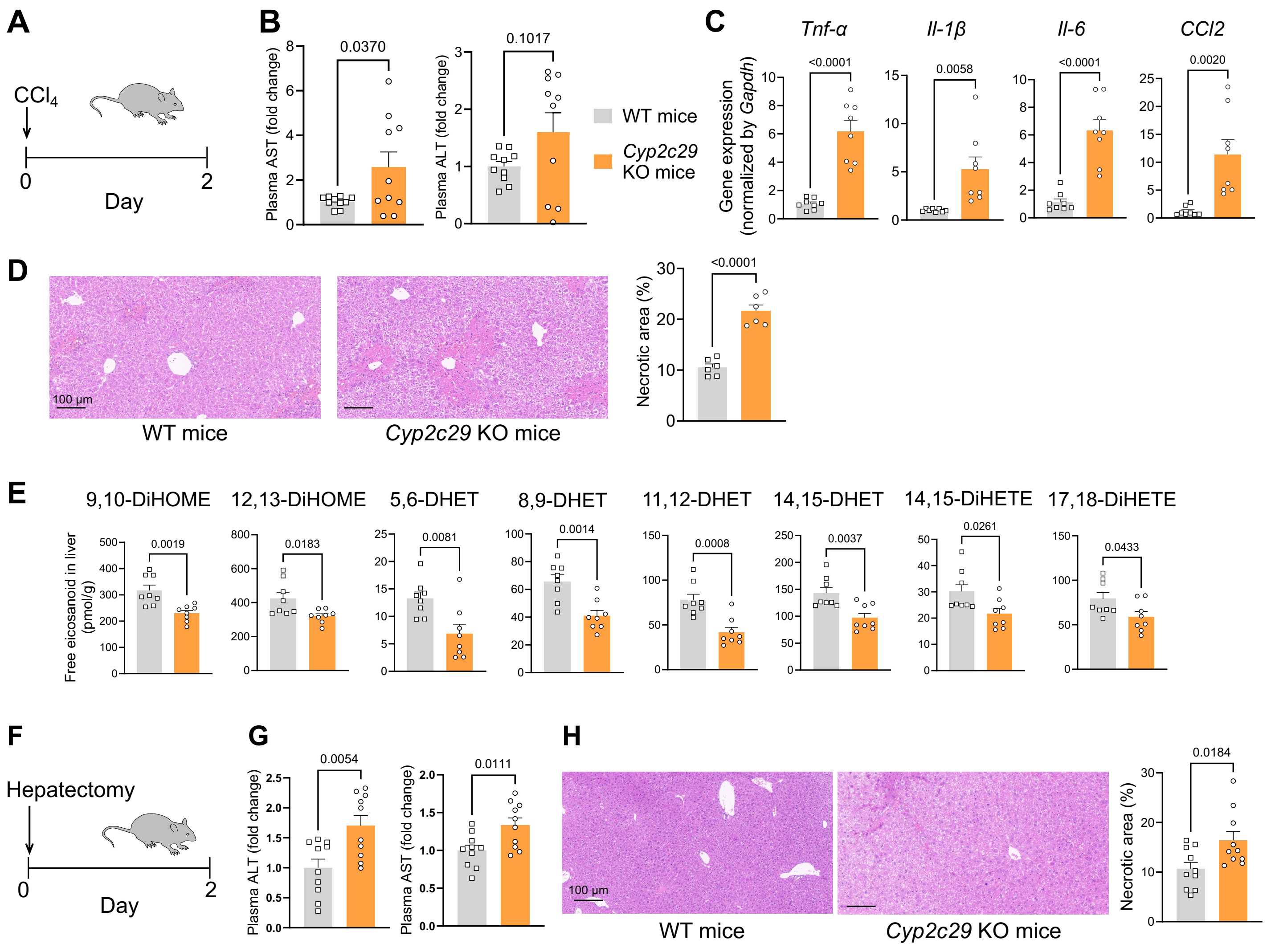

**Fig. 4. Genetic ablation of CYP monooxygenase exacerbates both chemically and surgically induced liver injury. A.** We treated WT mice or *Cyp2c29* KO mice with $CCl_4$ to induce liver injury. **B.** Plasma concentrations of AST and ALT are increased in *Cyp2c29* KO mice (n = 10 mice per group). **C.** Gene expression of pro-inflammatory mediators is increased in the liver of *Cyp2c29* KO mice (n = 8 mice per group). **D.** H&E histology shows that *Cyp2c29* KO mice have more severe liver damage than WT mice (n = 6 mice per group). **E.** Concentrations of CYP-derived eicosanoids are decreased in the liver of *Cyp2c29* KO mice compared to WT mice (n = 8 mice per group). **F.** We performed hepatectomy surgery in WT mice or *Cyp2c29* KO mice to induce liver injury. **G.** Plasma concentrations of AST and ALT are increased in *Cyp2c29* KO mice (n = 10 mice per group). **H.** Liver histology shows that *Cyp2c29* KO mice have more severe liver damage than WT mice (n = 10 mice per group). The data are mean ± SEM.

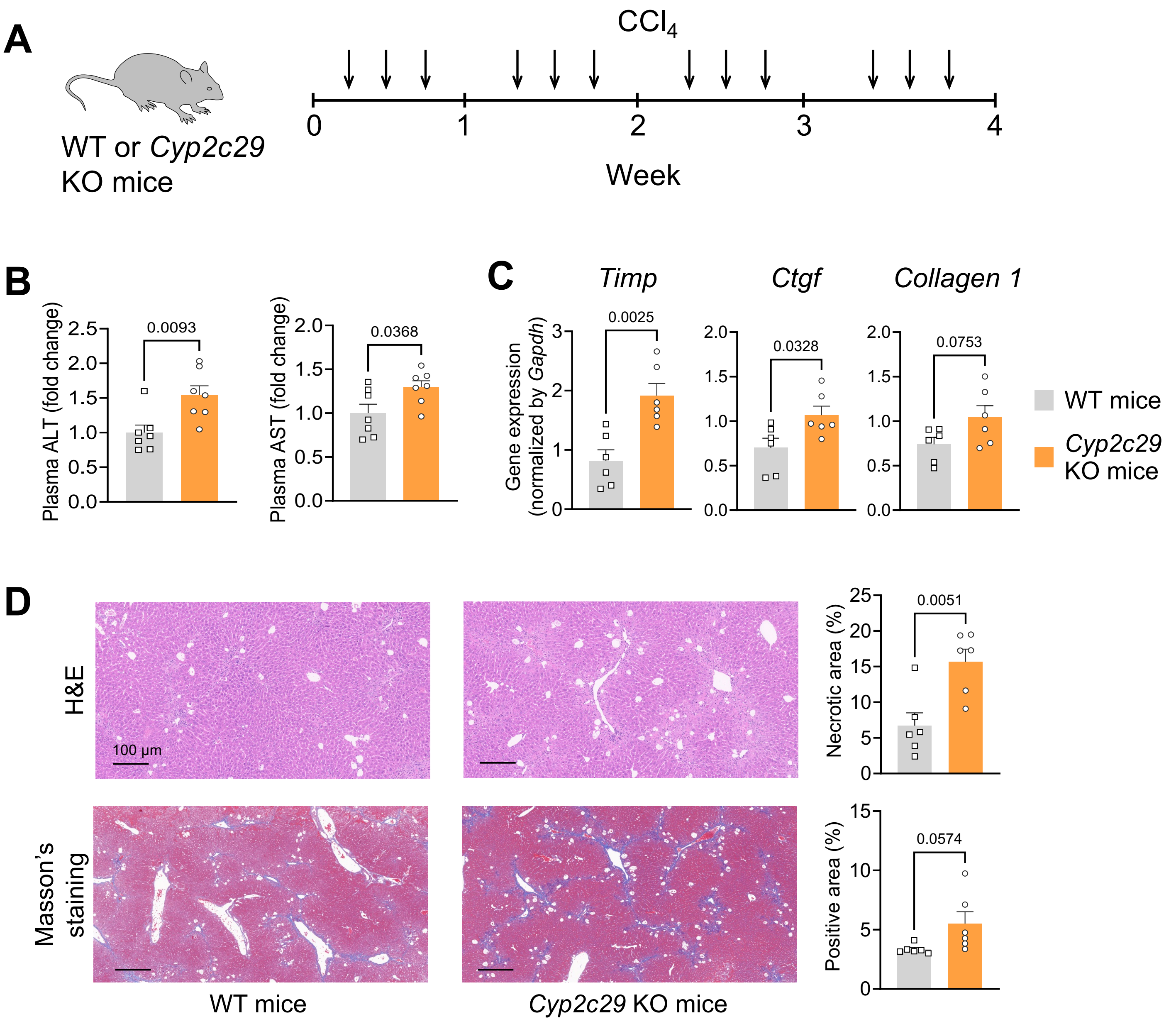

**Fig. 5. Genetic ablation of CYP monooxygenase exacerbates chronic $CCl_4$-induced liver fibrosis. A.** We treated WT mice or *Cyp2c29* KO mice with repeated i.p. injections of low-dose $CCl_4$ to induce liver fibrosis. **B.** Plasma concentrations of AST and ALT are increased in *Cyp2c29* KO mice (n = 7 mice per group). **C.** Gene expression of fibrosis markers is increased in the liver of *Cyp2c29* KO mice (n = 6 mice per group). **D.** H&E and Masson's staining show that *Cyp2c29* KO mice have more severe liver damage and fibrosis than WT mice (n = 6 mice per group). The data are mean ± SEM.

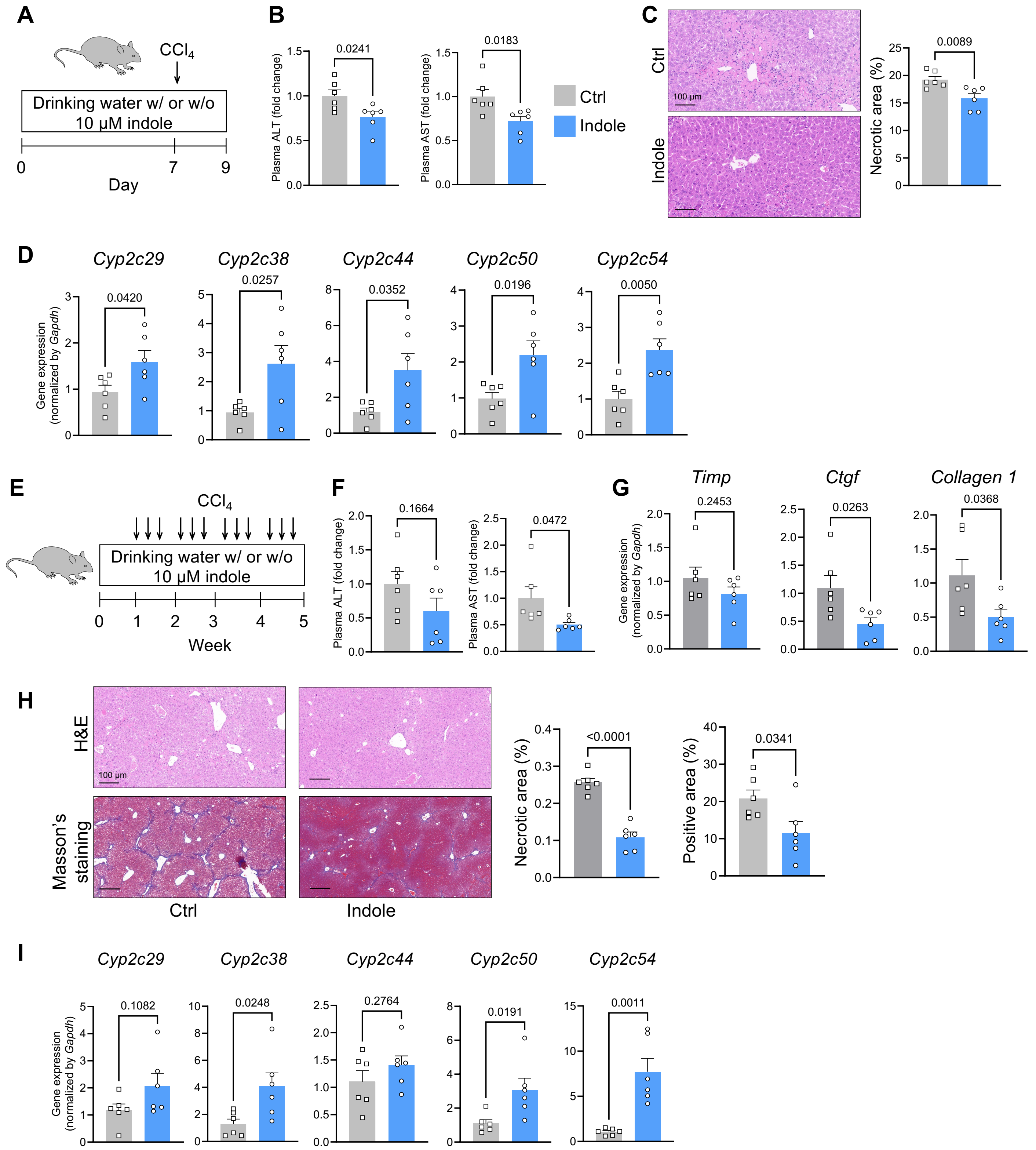

**Fig. 6. Indole treatment attenuates $CCl_4$-induced liver injury and fibrosis. A.** We treated SPF C57BL/6 mice with indole and then stimulated the mice with $CCl_4$ to induce liver injury. **B.** Indole treatment reduces plasma concentrations of AST and ALT (n = 6 mice per group). **C.** H&E histology shows that indole treatment attenuates liver damage in mice (n = 6 mice per group). **D.** Indole increases the expression of *Cyp2c* monooxygenases in the liver (n = 6 mice per group). **E.** We treated SPF C57BL/6 mice with indole and stimulated the mice with repeated i.p. injections of low-dose $CCl_4$ to induce liver fibrosis. **F.** Indole reduces plasma concentrations of AST and ALT (n = 6 mice per group). **G.** Indole decreases gene expression of fibrosis markers in the liver (n = 6 mice per group). **H.** H&E and Masson's staining show that indole treatment attenuated liver damage and fibrosis (n = 6 mice per group). **I.** Indole increases the expression of *Cyp2c* monooxygenases in the liver (n = 6 mice per group). The data are mean ± SEM.

**Table S1. Previous studies of gut microbiota on chemically and surgically induced liver injury and fibrosis** (This table is modified from *Am J Physiol Gastrointest Liver Physiol.* 2019, 316(5):G563–G573, PMID: 30767680)

| **Liver disease** | **Experimental model** | **Animal strain** | **Disease development (GF *vs.* Conventional)** | **Reference** |
|---|---|---|---|---|
| Liver injury | Butter yellow | Fischer rats | Exacerbated in GF | *Lab Invest* 16: 847–857, 1967 (PMID: 4168350) |
| Liver injury | Partial hepatectomy | BALB/c mice | Exacerbated in GF | *Hepatology* 11: 916 –922, 1990 (PMID: 2194922) |
| Liver injury | Chlortetracycline | ICR mice | Exacerbated in GF | *Biochem Pharmacol* 15: 1093–1104, 1966 (PMID: 5973157) |
| Liver injury | DMA plus sodium nitrite | Carworth Farms Swiss Webster mice | Exacerbated in GF | *Proc Soc Exp Biol Med* 140: 1073–1076, 1972 (PMID: 5039367) |
| Liver injury | DMN or DMA plus sodium nitrite | Wistar rats | Exacerbated in GF | *Cancer Res* 43: 2942–2946, 1983 (PMID: 6850604) |
| Liver fibrosis/cirrhosis | $CCl_4$ | ICR mice | Exacerbated in GF | *Armed Services Technical Information Agency*, 1963, 15 pp. (Publ. AD-403868) |
| Liver injury and fibrosis | Thioacetamide (TAA) or $CCl_4$ | C57BL/6 mice | Exacerbated in GF | *FASEB J* 29: 1043–1055, 2015 (PMID: 25466902) |
| Alcohol-induced liver injury | Single gavage of alcohol | C57BL/6 mice | Exacerbated in GF | *Alcohol Clin Exp Res* 39:2313-23, 2015 (PMID: 26556636) |

**Table S2. LC-MS/MS analysis of free eicosanoids in the liver of specific pathogen-free (SPF) mice and germ-free (GF) C57BL/6 mice**

| Metabolite | SPF mice (pmol/g in tissue) | | GF mice (pmol/g in tissue) | | P value | $Log_2FC$ * |
|---|---|---|---|---|---|---|
| | Average | SEM | Average | SEM | | |
| 10,11-DiHDPA | 39.544 | 4.851 | 22.128 | 2.034 | 0.004 | 0.838 |
| 10,11-EpDPA | 59.603 | 11.100 | 32.321 | 4.038 | 0.037 | 0.883 |
| 11,12-DHET | 210.201 | 15.092 | 136.102 | 14.269 | 0.002 | 0.627 |
| 11,12-DiHETE | 5.030 | 0.728 | 3.326 | 0.255 | 0.045 | 0.597 |
| 11,12-EET | 47.722 | 5.671 | 34.112 | 4.835 | 0.085 | 0.484 |
| 11,12-EpETE | 25.624 | 3.792 | 16.475 | 1.457 | 0.041 | 0.637 |
| 11-dehydro-TXB2 | 0.307 | 0.025 | 0.310 | 0.044 | 0.957 | -0.015 |
| 11-HETE | 89.344 | 7.206 | 81.343 | 8.454 | 0.477 | 0.135 |
| 12,13-DHOME | 1238.765 | 135.054 | 1407.238 | 142.348 | 0.400 | -0.184 |
| 12,13-EpOME | 221.778 | 28.194 | 148.633 | 12.948 | 0.033 | 0.577 |
| 12-HETE | 520.935 | 65.636 | 403.417 | 98.566 | 0.325 | 0.369 |
| 13,14-DiHDPA | 117.705 | 8.019 | 87.021 | 8.376 | 0.015 | 0.436 |
| 13,14-EpDPA | 36.601 | 4.751 | 23.533 | 3.048 | 0.034 | 0.637 |
| 13-HODE | 2103.799 | 192.425 | 1586.857 | 87.279 | 0.027 | 0.407 |
| 14,15-DHET | 373.949 | 19.855 | 277.249 | 26.483 | 0.008 | 0.432 |
| 14,15-DiHETE | 423.174 | 41.400 | 273.908 | 20.693 | 0.005 | 0.628 |
| 14,15-EET | 127.314 | 13.395 | 90.393 | 10.544 | 0.044 | 0.494 |
| 14,15-EpETE | 22.962 | 3.397 | 14.506 | 1.301 | 0.036 | 0.663 |
| 15-HETE | 164.146 | 15.915 | 105.311 | 7.365 | 0.004 | 0.640 |
| 15keto-PGF2a | 5808.962 | 519.344 | 5880.440 | 506.350 | 0.923 | -0.018 |
| 16,17-DiHDPA | 181.205 | 9.484 | 156.140 | 13.460 | 0.138 | 0.215 |
| 16,17-EpDPA | 42.894 | 6.666 | 24.989 | 2.738 | 0.026 | 0.780 |
| 17,18-DiHETE | 1554.111 | 136.769 | 1348.810 | 98.946 | 0.245 | 0.204 |
| 17,18-EpETE | 49.261 | 6.120 | 39.132 | 4.734 | 0.210 | 0.332 |
| 17-HDHA | 81.981 | 8.585 | 55.316 | 4.231 | 0.013 | 0.568 |
| 18-HEPE | 314.487 | 39.053 | 152.790 | 6.442 | 0.001 | 1.042 |
| 19,20-DiHDPA | 781.698 | 37.094 | 988.011 | 84.012 | 0.031 | -0.338 |
| 19,20-EpDPE | 541.848 | 117.510 | 199.338 | 19.746 | 0.012 | 1.443 |
| 19-HETE | 10.433 | 1.349 | 9.102 | 0.585 | 0.391 | 0.197 |
| 20-carboxy-AA | 21.844 | 2.288 | 40.622 | 5.517 | 0.004 | -0.895 |
| 20-HEPE | 39.585 | 2.538 | 62.122 | 4.079 | 0.000 | -0.650 |
| 20-HETE | 5.131 | 0.885 | 6.487 | 1.076 | 0.338 | -0.338 |
| 22-HDoHE | 12.334 | 1.352 | 11.734 | 1.323 | 0.755 | 0.072 |
| 5,6-DHET | 39.441 | 3.679 | 27.234 | 3.246 | 0.022 | 0.534 |
| 5-HETE | 97.044 | 13.031 | 42.485 | 3.883 | 0.001 | 1.192 |
| 6ketoPGF1a | 34.544 | 7.711 | 47.539 | 6.185 | 0.208 | -0.461 |
| 7,8-DiHDPA | 40.485 | 3.544 | 24.151 | 2.334 | 0.001 | 0.745 |
| 7,8-EpDPA | 117.933 | 23.006 | 56.282 | 6.624 | 0.022 | 1.067 |

| | | | | | | |
|---|---|---|---|---|---|---|
| 8,9-DHET | 138.077 | 12.106 | 73.812 | 8.153 | 0.000 | 0.904 |
| 8,9-EET | 149.318 | 22.161 | 68.732 | 10.385 | 0.005 | 1.119 |
| 8-HETE | 323.868 | 27.892 | 233.137 | 23.786 | 0.023 | 0.474 |
| 8isoPGF2a | 2.862 | 0.180 | 2.152 | 0.190 | 0.013 | 0.412 |
| 9,10-DHOME | 482.234 | 81.774 | 300.773 | 28.086 | 0.056 | 0.681 |
| 9,10-EpOME | 470.742 | 76.890 | 271.475 | 18.652 | 0.025 | 0.794 |
| 9-HODE | 2625.735 | 236.397 | 1953.515 | 133.248 | 0.025 | 0.427 |
| LTB4 | 1.698 | 0.275 | 1.090 | 0.159 | 0.076 | 0.639 |
| PGD2 | 100.138 | 6.363 | 108.885 | 10.889 | 0.487 | -0.121 |
| PGD3 | 5.850 | 0.577 | 8.906 | 0.848 | 0.007 | -0.606 |
| PGE2 | 69.294 | 4.174 | 127.771 | 21.451 | 0.011 | -0.883 |
| PGE3 | 14.020 | 1.365 | 26.364 | 4.709 | 0.016 | -0.911 |
| PGF2a | 111.537 | 11.982 | 127.015 | 6.785 | 0.285 | -0.188 |
| TXB2 | 55.097 | 11.710 | 39.565 | 4.062 | 0.240 | 0.478 |

* FC: fold of change, representing the level of the lipid metabolite in the SPF mice to that of the GF mice.

**Table S3. LC-MS/MS analysis of total eicosanoids in the liver of specific pathogen-free (SPF) mice and germ-free (GF) C57BL/6 mice**

| Metabolite | SPF mice (pmol/g in tissue) | | GF mice (pmol/g in tissue) | | P value | $Log_2FC$ |
|---|---|---|---|---|---|---|
| | Average | SEM | Average | SEM | | |
| 10,11-DiHDPA | 58.213 | 8.751 | 31.443 | 3.177 | 0.011 | 0.889 |
| 10,11-EpDPA | 495.989 | 140.513 | 184.023 | 20.720 | 0.048 | 1.430 |
| 11,12-DHET | 393.133 | 37.779 | 223.293 | 21.143 | 0.001 | 0.816 |
| 11,12-DiHETE | 6.730 | 0.871 | 4.135 | 0.373 | 0.015 | 0.703 |
| 11,12-EET | 520.542 | 139.345 | 199.214 | 21.277 | 0.041 | 1.386 |
| 11,12-EpETE | 96.051 | 25.133 | 42.525 | 4.736 | 0.058 | 1.176 |
| 11-dehydro-TXB2 | 0.489 | 0.051 | 0.267 | 0.016 | 0.107 | 0.872 |
| 11-HETE | 289.874 | 65.370 | 150.156 | 13.802 | 0.058 | 0.949 |
| 12,13-DHOME | 1141.767 | 114.470 | 1329.727 | 157.251 | 0.339 | -0.220 |
| 12,13-EpOME | 476.538 | 98.959 | 301.478 | 37.202 | 0.125 | 0.661 |
| 12-HETE | 822.102 | 129.561 | 517.768 | 111.525 | 0.092 | 0.667 |
| 13,14-DiHDPA | 182.739 | 20.340 | 119.215 | 11.367 | 0.015 | 0.616 |
| 13,14-EpDPA | 247.144 | 63.151 | 117.074 | 13.344 | 0.067 | 1.078 |
| 13-HODE | 3524.725 | 751.665 | 1821.067 | 154.230 | 0.045 | 0.953 |
| 14,15-DHET | 399.991 | 28.379 | 299.233 | 30.777 | 0.025 | 0.419 |
| 14,15-DiHETE | 473.444 | 50.076 | 306.716 | 28.943 | 0.010 | 0.626 |
| 14,15-EET | 1180.036 | 229.917 | 589.533 | 57.752 | 0.026 | 1.001 |
| 14,15-EpETE | 83.833 | 16.551 | 47.013 | 5.244 | 0.054 | 0.835 |
| 15-HETE | 1023.684 | 303.611 | 277.690 | 19.993 | 0.029 | 1.882 |
| 15keto-PGF2a | 5274.332 | 488.758 | 4899.947 | 460.253 | 0.585 | 0.106 |
| 16,17-DiHDPA | 262.185 | 21.683 | 214.681 | 19.718 | 0.122 | 0.288 |
| 16,17-EpDPA | 320.508 | 69.000 | 179.562 | 18.320 | 0.072 | 0.836 |
| 17,18-DiHETE | 1521.309 | 141.732 | 1313.709 | 110.934 | 0.268 | 0.212 |
| 17,18-EpETE | 124.261 | 21.041 | 88.597 | 12.080 | 0.166 | 0.488 |
| 17-HDHA | 466.425 | 124.541 | 199.917 | 16.939 | 0.055 | 1.222 |
| 18-HEPE | 693.374 | 172.010 | 237.939 | 19.738 | 0.020 | 1.543 |
| 19,20-DiHDPA | 769.820 | 54.898 | 1009.147 | 98.487 | 0.042 | -0.391 |
| 19,20-EpDPE | 2546.756 | 675.028 | 1295.345 | 136.154 | 0.096 | 0.975 |
| 19-HETE | 30.295 | 3.637 | 24.012 | 2.118 | 0.160 | 0.335 |
| 20-carboxy-AA | 16.725 | 0.873 | 25.737 | 2.449 | 0.002 | -0.622 |
| 20-HEPE | 68.435 | 4.526 | 105.683 | 8.169 | 0.001 | -0.627 |
| 20-HETE | 24.546 | 2.051 | 27.369 | 3.076 | 0.447 | -0.157 |
| 22-HDoHE | 30.715 | 6.077 | 22.938 | 2.224 | 0.259 | 0.421 |
| 5,6-DHET | 143.114 | 24.629 | 67.657 | 7.922 | 0.011 | 1.081 |
| 5-HETE | 762.401 | 216.157 | 221.872 | 15.204 | 0.027 | 1.781 |
| 6ketoPGF1a | 21.838 | 3.486 | 26.436 | 3.491 | 0.363 | -0.276 |
| 7,8-DiHDPA | 162.833 | 13.553 | 87.073 | 9.122 | 0.000 | 0.903 |
| 7,8-EpDPA | 990.887 | 298.403 | 309.078 | 36.559 | 0.042 | 1.681 |

| | | | | | | |
|---|---|---|---|---|---|---|
| 8,9-DHET | 285.880 | 30.574 | 130.855 | 13.237 | 0.000 | 1.127 |
| 8,9-EET | 1256.903 | 381.968 | 350.617 | 38.082 | 0.035 | 1.842 |
| 8-HETE | 2485.771 | 515.302 | 1185.018 | 97.857 | 0.027 | 1.069 |
| 8isoPGF2a | 2.352 | 0.145 | 1.741 | 0.138 | 0.006 | 0.434 |
| 9,10-DHOME | 475.563 | 79.508 | 308.021 | 36.174 | 0.077 | 0.627 |
| 9,10-EpOME | 1411.162 | 360.825 | 627.855 | 69.947 | 0.054 | 1.168 |
| 9-HODE | 4207.950 | 889.917 | 2204.181 | 167.925 | 0.046 | 0.933 |
| LTB4 | 3.723 | 1.210 | 1.225 | 0.182 | 0.064 | 1.604 |
| PGD2 | 126.679 | 12.350 | 103.259 | 10.289 | 0.164 | 0.295 |
| PGD3 | 4.999 | 0.504 | 7.926 | 0.897 | 0.008 | -0.665 |
| PGE2 | 66.288 | 3.568 | 119.181 | 22.863 | 0.027 | -0.846 |
| PGE3 | 11.589 | 1.158 | 19.925 | 3.317 | 0.023 | -0.782 |
| PGF2a | 81.264 | 7.581 | 91.382 | 6.218 | 0.319 | -0.169 |
| TXB2 | 42.474 | 6.780 | 28.642 | 3.120 | 0.087 | 0.569 |

**Table S4. LC-MS/MS analysis of free eicosanoids in the liver of germ-free (GF) C57BL/6 mice mono-colonized with WT *Bt* and *Bt* Δ1492**

| Metabolite | GF mice mono-colonized with WT *Bt* (pmol/g in tissue) | | GF mice mono-colonized with *Bt* Δ1492 (pmol/g in tissue) | | P value | $Log_2FC$ * |
|---|---|---|---|---|---|---|
| | Average | SEM | Average | SEM | | |
| 10,11-DiHDPA | 3.501 | 0.319 | 2.920 | 0.273 | 0.184 | 0.262 |
| 10,11-EpDPA | 3.763 | 0.249 | 3.713 | 0.295 | 0.897 | 0.020 |
| 11,12-DHET | 34.595 | 3.973 | 26.532 | 2.926 | 0.120 | 0.383 |
| 11,12-DiHETE | 2.559 | 0.358 | 1.902 | 0.234 | 0.142 | 0.428 |
| 11,12-EET | 3.248 | 0.201 | 3.306 | 0.175 | 0.831 | -0.025 |
| 11,12-EpETE | 1.854 | 0.138 | 1.867 | 0.161 | 0.953 | -0.010 |
| 11-HETE | 40.571 | 4.197 | 35.727 | 4.115 | 0.421 | 0.183 |
| 12,13-DHOME | 278.582 | 10.958 | 229.758 | 15.921 | **0.021** | 0.278 |
| 12,13-EpOME | 25.658 | 1.793 | 23.364 | 1.989 | 0.403 | 0.135 |
| 12-HETE | 96.557 | 10.818 | 101.897 | 21.369 | 0.826 | -0.078 |
| 13,14-DiHDPA | 13.684 | 0.884 | 12.248 | 0.618 | 0.200 | 0.160 |
| 13,14-EpDPA | 3.225 | 0.208 | 3.209 | 0.259 | 0.962 | 0.007 |
| 13-HODE | 1248.507 | 45.770 | 1104.550 | 87.708 | 0.163 | 0.177 |
| 14,15-DHET | 75.174 | 7.126 | 62.229 | 5.859 | 0.178 | 0.273 |
| 14,15-DiHETE | 219.856 | 26.267 | 176.951 | 18.075 | 0.195 | 0.313 |
| 14,15-EET | 6.197 | 0.422 | 5.958 | 0.347 | 0.667 | 0.057 |
| 14,15-EpETE | 2.148 | 0.132 | 2.057 | 0.127 | 0.626 | 0.063 |
| 15-HETE | 31.810 | 2.555 | 26.641 | 2.005 | 0.129 | 0.256 |
| 16,17-DiHDPA | 29.499 | 1.480 | 25.892 | 1.009 | 0.059 | 0.188 |
| 16,17-EpDPA | 3.058 | 0.194 | 2.864 | 0.178 | 0.469 | 0.095 |
| 17,18-DiHETE | 531.123 | 29.663 | 487.770 | 27.592 | 0.299 | 0.123 |
| 17,18-EpETE | 6.176 | 0.416 | 5.653 | 0.390 | 0.371 | 0.128 |
| 17-HDHA | 50.418 | 4.711 | 40.489 | 5.021 | 0.166 | 0.316 |
| 18-HEPE | 62.587 | 3.495 | 49.050 | 2.430 | **0.005** | 0.352 |
| 19,20-DiHDPA | 112.068 | 4.673 | 103.143 | 4.194 | 0.172 | 0.120 |
| 19,20-EpDPE | 39.692 | 3.338 | 33.190 | 3.139 | 0.173 | 0.258 |
| 19-HETE | 13.207 | 1.424 | 10.860 | 1.165 | 0.218 | 0.282 |
| 20-carboxy-AA | 2.025 | 0.132 | 2.395 | 0.159 | 0.092 | -0.242 |
| 20-HEPE | 23.743 | 1.057 | 21.257 | 0.907 | 0.091 | 0.160 |
| 20-HETE | 6.875 | 0.759 | 5.206 | 0.467 | 0.077 | 0.401 |
| 22-HDoHE | 7.247 | 0.857 | 5.515 | 0.476 | 0.094 | 0.394 |
| 5,6-DHET | 5.789 | 0.742 | 4.222 | 0.590 | 0.116 | 0.455 |
| 5-HETE | 16.587 | 1.228 | 12.822 | 1.209 | **0.042** | 0.371 |
| 6ketoPGF1a | 4.138 | 0.542 | 3.574 | 0.377 | 0.404 | 0.211 |
| 7,8-DiHDPA | 7.170 | 0.850 | 6.284 | 0.625 | 0.412 | 0.190 |
| 7,8-EpDPA | 5.808 | 0.402 | 5.469 | 0.457 | 0.584 | 0.087 |
| 8,9-DHET | 15.783 | 2.006 | 11.895 | 1.634 | 0.150 | 0.408 |

| | | | | | | |
|---|---|---|---|---|---|---|
| 8,9-EET | 3.569 | 0.178 | 3.424 | 0.283 | 0.669 | 0.060 |
| 8-HETE | 65.463 | 5.596 | 50.995 | 5.645 | 0.085 | 0.360 |
| 8isoPGF2a | 1.123 | 0.154 | 1.109 | 0.119 | 0.943 | 0.018 |
| 9,10-DHOME | 119.003 | 11.530 | 83.558 | 10.453 | **0.035** | 0.510 |
| 9,10-EpOME | 50.320 | 3.677 | 48.428 | 5.240 | 0.771 | 0.055 |
| 9-HODE | 1046.432 | 39.616 | 934.073 | 72.686 | 0.191 | 0.164 |
| LTB4 | 0.862 | 0.110 | 0.966 | 0.076 | 0.451 | -0.163 |
| PGD2 | 72.082 | 8.343 | 64.481 | 7.812 | 0.514 | 0.161 |
| PGD3 | 33.308 | 2.925 | 29.810 | 3.294 | 0.438 | 0.160 |
| PGE2 | 28.087 | 3.093 | 24.852 | 1.973 | 0.390 | 0.176 |
| PGF2a | 48.536 | 6.951 | 47.511 | 5.561 | 0.910 | 0.031 |
| TXB2 | 10.560 | 1.342 | 9.704 | 1.228 | 0.644 | 0.122 |

* FC: fold of change, representing the level of the lipid metabolite in GF mice mono-colonized with WT *Bt* to that of the GF mice mono-colonized with *Bt* Δ1492.

**Table S5. LC-MS/MS analysis of total eicosanoids in the liver of germ-free (GF) C57BL/6 mice mono-colonized with WT *Bt* and *Bt* Δ1492**

| Metabolite | GF mice mono-colonized with WT *Bt* (pmol/g in tissue) | | GF mice mono-colonized with *Bt* Δ1492 (pmol/g in tissue) | | P value | $Log_2FC$ * |
|---|---|---|---|---|---|---|
| | Average | SEM | Average | SEM | | |
| 10,11-DiHDPA | 8.250 | 0.440 | 7.390 | 0.237 | 0.103 | 0.159 |
| 10,11-EpDPA | 171.341 | 4.027 | 149.781 | 3.616 | **0.001** | 0.194 |
| 11,12-DHET | 67.693 | 4.875 | 61.607 | 3.127 | 0.307 | 0.136 |
| 11,12-DiHETE | 3.045 | 0.344 | 2.407 | 0.211 | 0.131 | 0.339 |
| 11,12-EET | 223.924 | 7.475 | 198.816 | 5.000 | **0.012** | 0.172 |
| 11,12-EpETE | 9.604 | 0.484 | 7.724 | 0.430 | **0.009** | 0.314 |
| 11-HETE | 159.398 | 8.036 | 157.747 | 4.888 | 0.863 | 0.015 |
| 12,13-DHOME | 323.887 | 12.398 | 264.362 | 17.577 | **0.013** | 0.293 |
| 12,13-EpOME | 602.317 | 17.587 | 491.393 | 6.410 | **0.000** | 0.294 |
| 12-HETE | 41.563 | 4.344 | 44.562 | 6.667 | 0.711 | -0.100 |
| 13,14-DiHDPA | 45.597 | 2.210 | 42.920 | 0.967 | 0.282 | 0.087 |
| 13,14-EpDPA | 134.180 | 3.493 | 114.575 | 3.269 | **0.001** | 0.228 |
| 13-HODE | 2220.471 | 106.199 | 2051.314 | 97.955 | 0.257 | 0.114 |
| 14,15-DHET | 110.490 | 7.426 | 93.867 | 7.069 | 0.122 | 0.235 |
| 14,15-DiHETE | 252.527 | 26.963 | 204.871 | 17.931 | 0.158 | 0.302 |
| 14,15-EET | 664.777 | 25.535 | 590.658 | 12.480 | **0.018** | 0.171 |
| 14,15-EpETE | 52.392 | 1.820 | 42.711 | 2.176 | **0.003** | 0.295 |
| 15-HETE | 238.420 | 11.482 | 236.477 | 9.947 | 0.900 | 0.012 |
| 16,17-DiHDPA | 75.585 | 2.693 | 65.886 | 1.168 | **0.004** | 0.198 |
| 16,17-EpDPA | 253.710 | 8.892 | 207.221 | 7.169 | **0.001** | 0.292 |
| 17,18-DiHETE | 543.620 | 28.090 | 497.854 | 26.597 | 0.252 | 0.127 |
| 17,18-EpETE | 76.501 | 3.071 | 66.290 | 2.327 | **0.016** | 0.207 |
| 17-HDHA | 439.685 | 26.890 | 395.586 | 15.935 | 0.175 | 0.152 |
| 18-HEPE | 184.615 | 10.638 | 156.265 | 6.353 | **0.034** | 0.241 |
| 19,20-DiHDPA | 168.305 | 4.940 | 152.234 | 3.404 | **0.015** | 0.145 |
| 19,20-EpDPE | 1205.727 | 39.281 | 963.498 | 22.410 | **0.000** | 0.324 |
| 19-HETE | 54.799 | 1.858 | 52.871 | 2.228 | 0.515 | 0.052 |
| 20-carboxy-AA | 19.930 | 2.712 | 20.071 | 6.461 | 0.984 | -0.010 |
| 20-HEPE | 118.078 | 2.985 | 105.024 | 3.122 | **0.007** | 0.169 |
| 20-HETE | 43.844 | 1.319 | 41.538 | 1.290 | 0.227 | 0.078 |
| 22-HDoHE | 25.727 | 1.231 | 22.119 | 0.691 | **0.020** | 0.218 |
| 5,6-DHET | 52.459 | 2.678 | 52.752 | 2.688 | 0.939 | -0.008 |
| 5-HETE | 312.911 | 14.586 | 313.530 | 14.141 | 0.976 | -0.003 |
| 6ketoPGF1a | 3.372 | 0.505 | 2.821 | 0.248 | 0.339 | 0.258 |
| 7,8-DiHDPA | 58.686 | 2.735 | 59.225 | 1.341 | 0.861 | -0.013 |
| 7,8-EpDPA | 194.073 | 5.869 | 176.344 | 3.934 | **0.022** | 0.138 |
| 8,9-DHET | 81.614 | 4.001 | 79.009 | 3.999 | 0.651 | 0.047 |

| | | | | | | |
|---|---|---|---|---|---|---|
| 8,9-EET | 220.749 | 8.514 | 210.342 | 9.112 | 0.415 | 0.070 |
| 8-HETE | 302.012 | 17.210 | 295.261 | 18.582 | 0.793 | 0.033 |
| 8isoPGF2a | 1.406 | 0.115 | 1.540 | 0.096 | 0.382 | -0.131 |
| 9,10-DHOME | 150.712 | 12.030 | 111.665 | 10.502 | **0.025** | 0.433 |
| 9,10-EpOME | 562.118 | 17.007 | 478.320 | 14.379 | **0.001** | 0.233 |
| 9-HODE | 1804.877 | 81.540 | 1654.815 | 84.025 | 0.216 | 0.125 |
| LTB4 | 2.744 | 0.205 | 2.489 | 0.092 | 0.271 | 0.141 |
| PGD2 | 114.169 | 11.585 | 107.787 | 9.997 | 0.682 | 0.083 |
| PGD3 | 39.141 | 3.932 | 35.515 | 3.630 | 0.507 | 0.140 |
| PGE2 | 35.332 | 3.568 | 31.643 | 2.143 | 0.387 | 0.159 |
| PGF2a | 43.635 | 5.951 | 41.740 | 4.145 | 0.797 | 0.064 |
| TXB2 | 10.507 | 1.343 | 9.332 | 0.971 | 0.487 | 0.171 |

* FC: fold of change, representing the level of the lipid metabolite in GF mice mono-colonized with WT *Bt* to that of the GF mice mono-colonized with *Bt* Δ1492.

**Table S6. Sequences of primers used in qRT-PCR**

| Gene name | Forward | Reverse |
|---|---|---|
| **Mouse genes** | | |
| *Tnf-α* | CCCTCACACTCAGATCATCTTCT | GCTACGACGTGGGCTACAG |
| *Il-6* | TAGTCCTTCCTACCCCAATTTCC | TTGGTCCTTAGCCACTCCTTC |
| *Il-1β* | GCAACTGTTCCTGAACTCAACT | ATCTTTTGGGGTCCGTCAACT |
| *Ccl2/Mcp-1* | TTAAAAACCTGGATCGGAACCAA | GCATTAGCTTCAGATTTACGGGT |
| *Collagen 1* | GCTCCTCTTAGGGGCCACT | CCACGTCTCACCATTGGGG |
| *Ctgf* | GGGCCTCTTCTGCGATTTC | ATCCAGGCAAGTGCATTGGTA |
| *Timp* | CTTGGTTCCCTGGCGTACTC | ACCTGATCCGTCCACAAACAG |
| *Cyp2c29* | ATCTGGTCGTGTTCCTAGCG | AGTAGGCTTTGAGCCCAAATAC |
| *Cyp2c37* | AATGGAATGGGCCTTGCA | GCAACGTGCTTCTTCTTGAACG |
| *Cyp2c38* | CACGGCCCATTGTTGTATTGC | TGAGTGTGAAACGTCTTGTCTCT |
| *Cyp2c39* | GAGGAAGCATTCCAATGGTAGAA | TGTGAAGCGCCTAATCTCTTTC |
| *Cyp2c44* | GCTGCCCTATACAGATGCCG | GTGACGCTAAGAGTTGCCCA |
| *Cyp2c40* | GGCTCACAGCCTATTGTGGTA | TCAAAAACCGGAATCCTTCCTC |
| *Cyp2c50* | ACTGTGGTGTTGCATGGATATG | GAGAAGCGCCTTGTGTTTTTC |
| *Cyp2c54* | AGACAGAGCTATGAAAGAGGGAA | GTGAGAAGTGCCTCGTGTTTT |
| *Cyp2c55* | AATGATCTGGGGGTGATTTTCAG | GCGATCCTCGATGCTCCTC |
| *Cyp2c65* | ACAACCCAAAGACGGAGTTTAC | CTGTCCCAGCAGCAAACAGAT |
| *Cyp2c70* | AGTATGGCCCTGTGTTTACTGT | GCCTTGGCTGGTTCTACTGAG |
| *Cyp2j5* | TCTGGGAAGCACTCCATCTCA | CCCTGGTGGGTAGTTTTTGG |
| *Cyp2j6* | TTAGCCACGATCTGGGCAG | CTGGGGGATAGTTCTTGGGG |
| *Cyp2j8* | GCTACTATAGGTTGCCTGGTG | CCCAAATGCAGATGTGACAC |
| *Cyp2j9* | TGGCTGATTTCCTCAAAAACCG | ACTGCTGAAGGGATAGGTGGG |
| *Cyp2j13* | AGCTGCTGTCACCTTCCTTTT | TGCGTCAAGTCCATCTGGAAC |
| *16S rRNA* | TCGTCGGCAGCGTCAGATGTGTATAA GAGACAGCCTACGGGNGGCWGCAG | GTCTCGTGGGCTCGGAGATGTGTATAA GAGACAGGACTACHVGGGTATCTAATCC |
| *Gapdh* | AGGTCGGTGTGAACGGATTTG | TGTAGACCATGTAGTTGAGGTCA |
| **Human genes** | | |
| *CYP2C19* | GGAAAACGGA TTTGTGTGGGA | GGTCCTTTGGGTCAA TCAGAGA |
| *PXR* | TGATCTTGCACCACACCTCC | CCTGCTGTGCTCACCTATGA |
| *Actin* | AGCCTTGTAGGTACCCAACC | TCCCACTCACCTGAGGTGCTGAA |

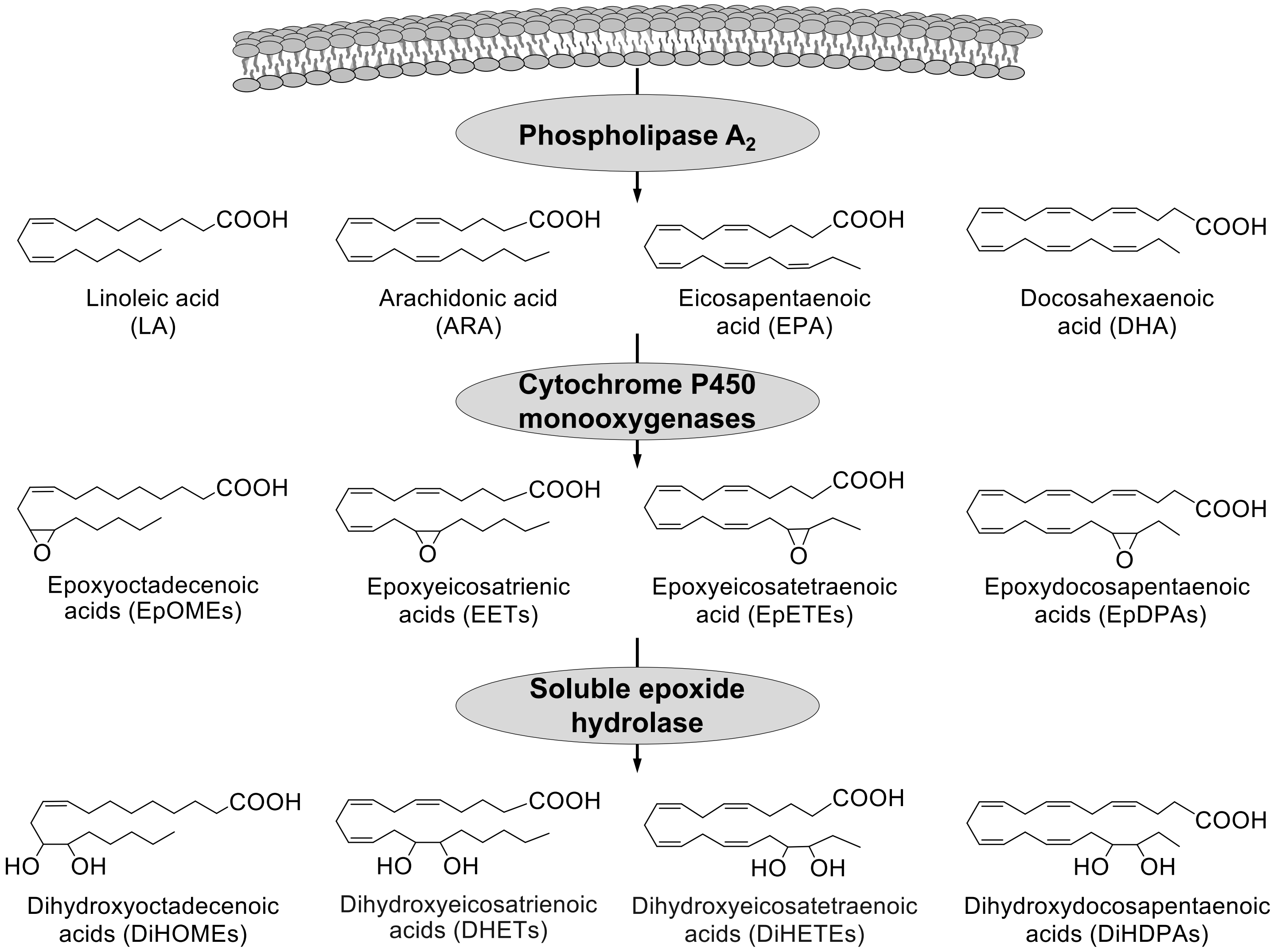

**Fig. S1. Biochemistry of the cytochrome P450 (CYP) eicosanoid pathway.** Upon cellular stimulation, long-chain polyunsaturated fatty acids (PUFAs), which are incorporated in cell membrane phospholipids, are released by enzymes such as phospholipase $A_2$ ($PLA_2$) to generate intracellular free PUFAs. The free PUFAs are metabolized by cytochrome P450 (CYP) monooxygenases, predominately CYP2C and CYP2J isoforms, forming fatty acid epoxides. Fatty acid epoxides are further metabolized by soluble epoxide hydrolase (sEH), generating the corresponding fatty acid diols.

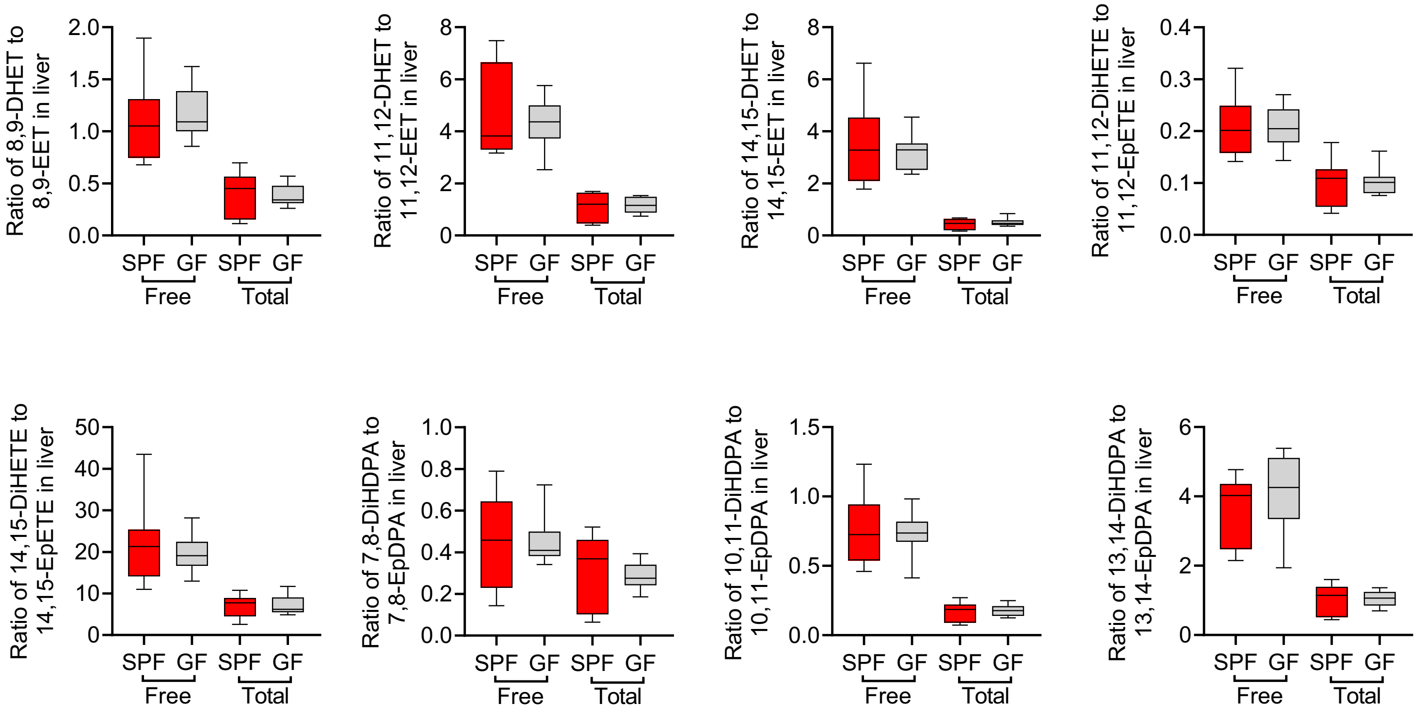

**Fig. S2. The ratio of fatty acid epoxide to fatty acid diol, which is a surrogate marker for the activity of soluble epoxide hydrolase (sEH), is not significantly altered in the liver of SPF mice versus GF mice.** SPF: specific pathogen-free mice, GF: germ-free mice, Free: free eicosanoids, Total: total eicosanoids (see details in Materials and Methods). n = 11-12 mice per group.

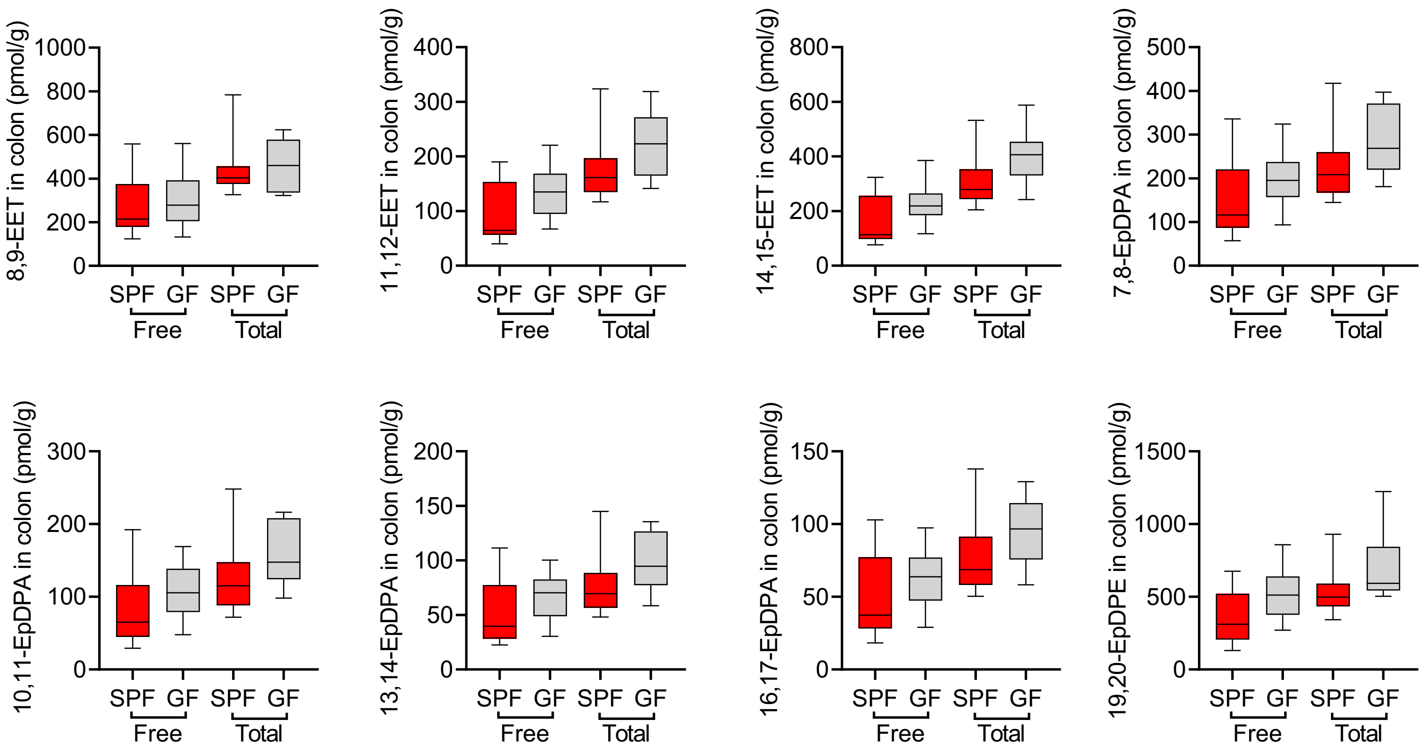

**Fig. S3. The concentrations of CYP-derived eicosanoids are not significantly altered in the colon tissues of GF mice compared with those of SPF mice.** n = 11-12 mice per group.

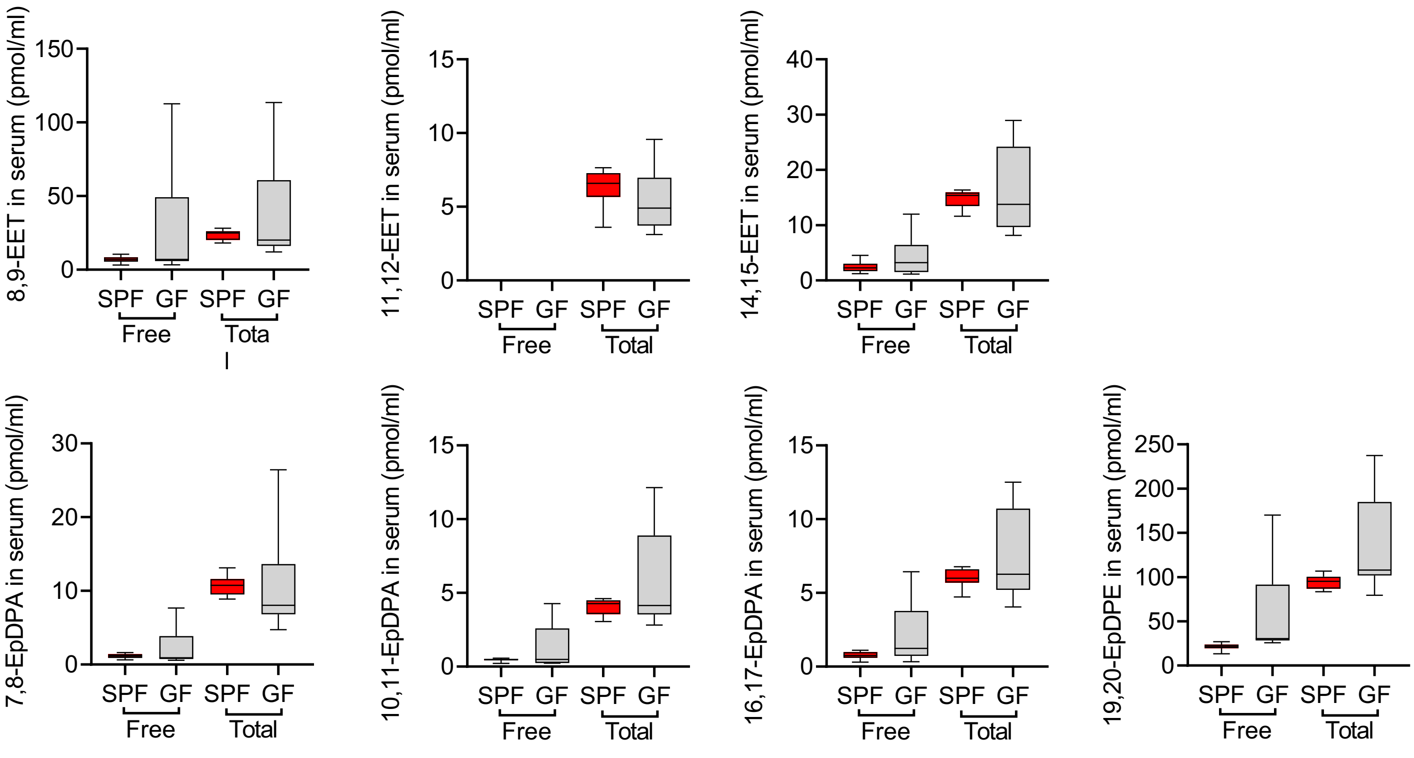

**Fig. S4. The concentrations of CYP-derived eicosanoids are not significantly altered in the serums of GF mice compared with those of SPF mice.** n = 11-12 mice per group.

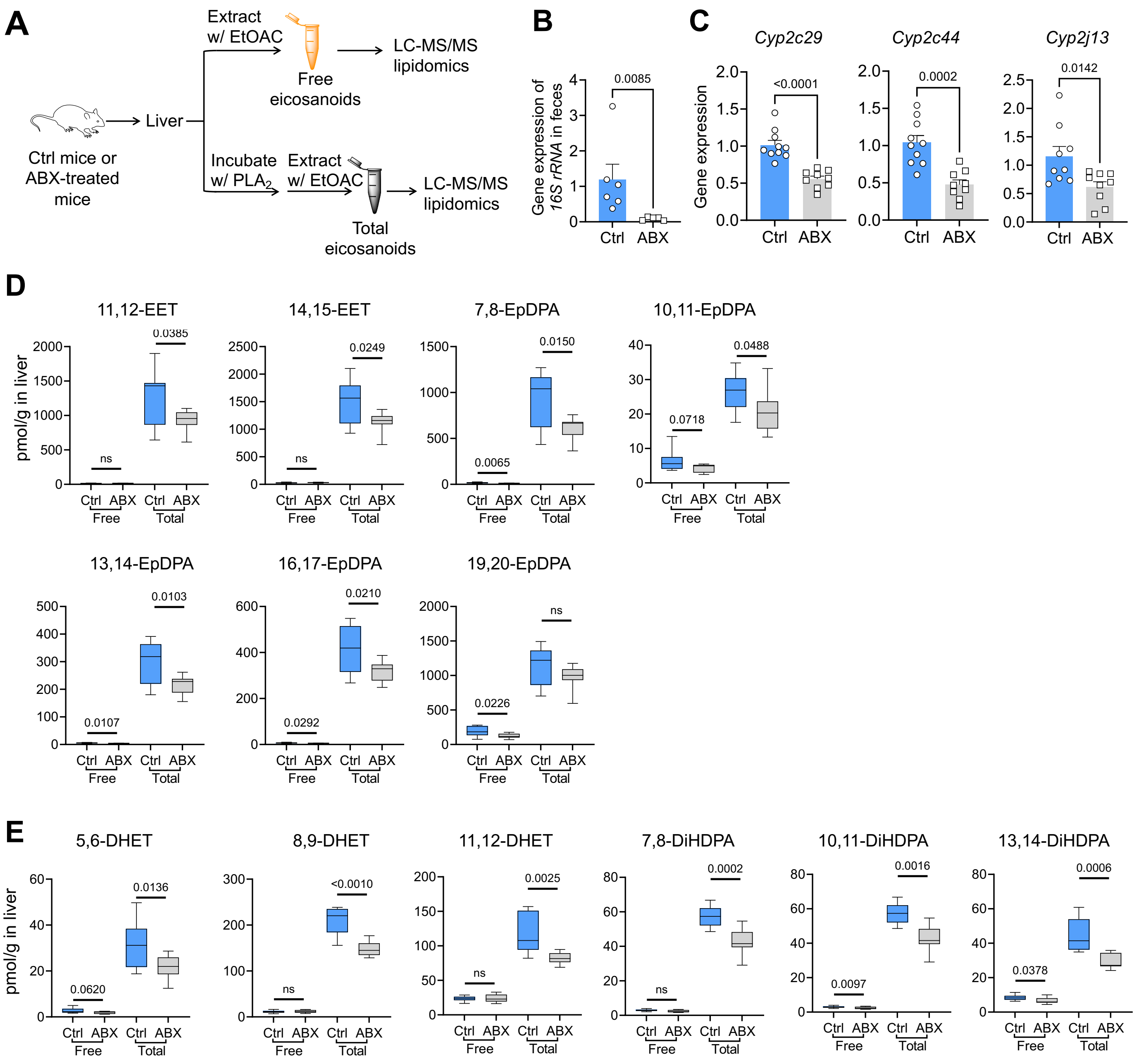

**Fig. S5. Compared with antibiotic-treated mice, the concentrations of CYP-derived eicosanoids and the expression of CYP monooxygenases are increased in the liver tissues of control mice. A.** Scheme of experiment. **B.** Antibiotic cocktail dramatically reduced total fecal microbial biomass in mice. **C.** Gene expression of CYP monooxygenases is increased in the livers of control mice compared to those of antibiotic-treated mice. The data are mean ± SEM. **D.** Concentrations of CYP-derived fatty acid epoxides are increased in the liver of control mice compared to those of antibiotic-treated mice. Free: free eicosanoids, Total: total eicosanoids. **E.** Concentrations of fatty acid diols, which are downstream metabolites of fatty acid epoxides, are increased in the liver of control mice compared to those of antibiotic-treated mice. n = 9-10 mice per group.

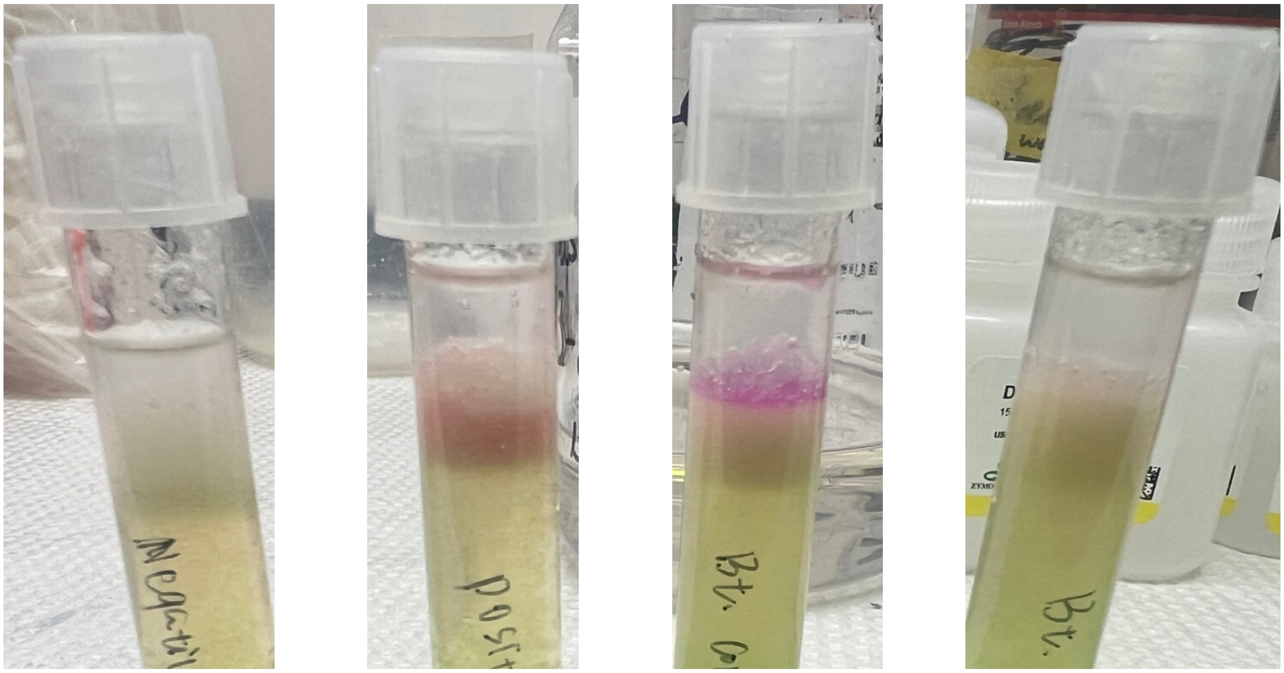

**Fig. S6.** Indole test using Ehrlich's reagent. Wild-type (WT) *B. thetaiotaomicron* (*Bt*) produces indole, whereas *Bt* Δ1492 lacks the ability to produce indole.

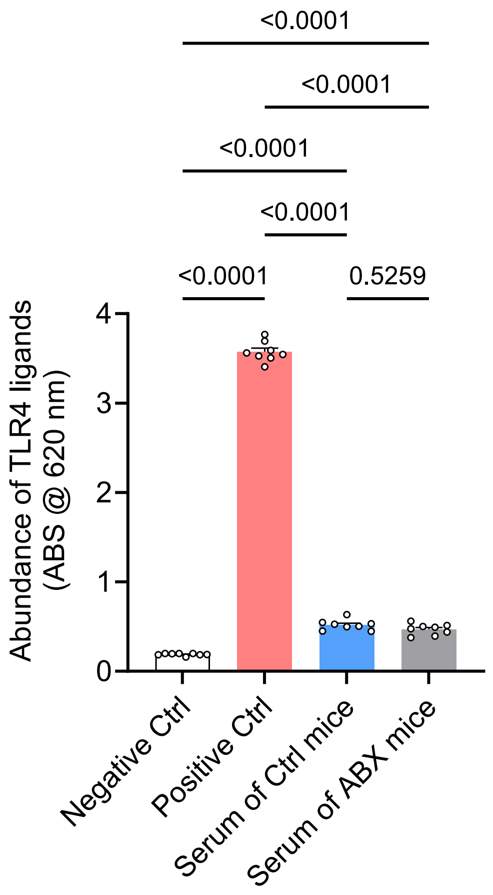

**Fig. S7. Levels of bacterial species in the circulation of control SPF C57BL/6 mice versus antibiotic-treated mice.** We employed a TLR4 reporter cell line-based assay to quantify the abundance of TLR4 ligands, such as LPS or specific bacteria, in the serum of mice. The levels of bacterial species in the circulation of both control and antibiotic-treated mice were comparable to those in the negative control (PBS buffer), indicating a minimal presence of bacterial species in the circulation of both mouse groups. Moreover, there was little difference observed between these two groups of mice in terms of the levels of bacterial species in the circulation. The data are mean ± SEM, n = 8 mice per group.

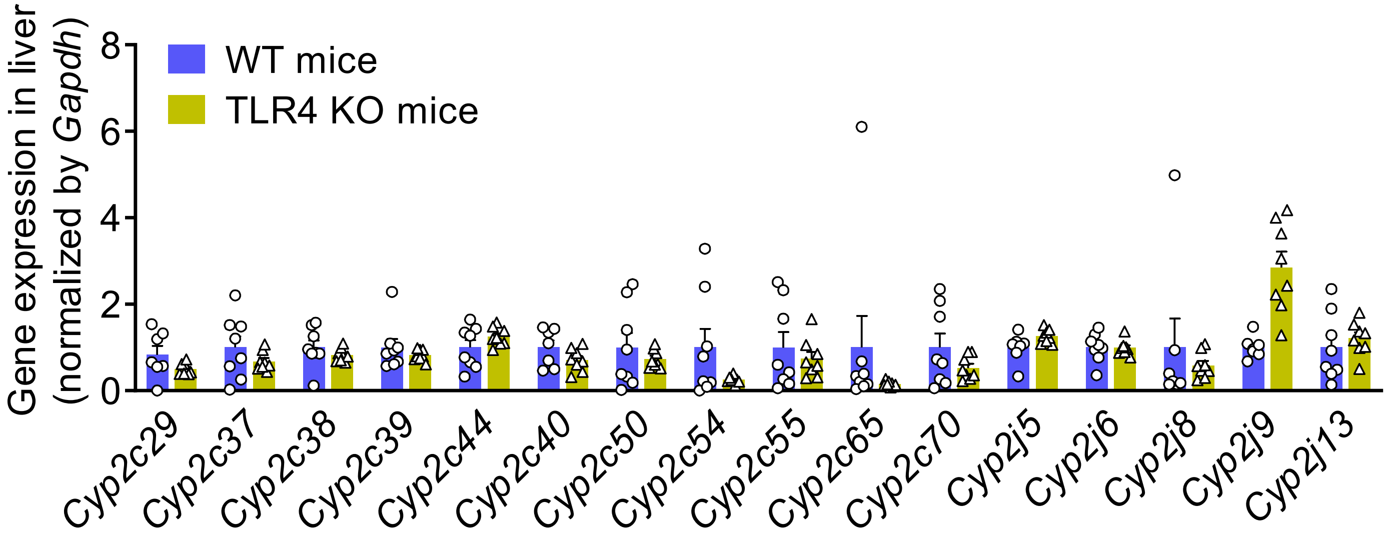

**Fig. S8. Gene expression of *Cyp2c/2j* monooxygenases is not significantly changed in the liver tissues of WT C57BL/6 mice compared to those of TLR4 KO mice.** The data are mean ± SEM, n = 6-8 mice per group.

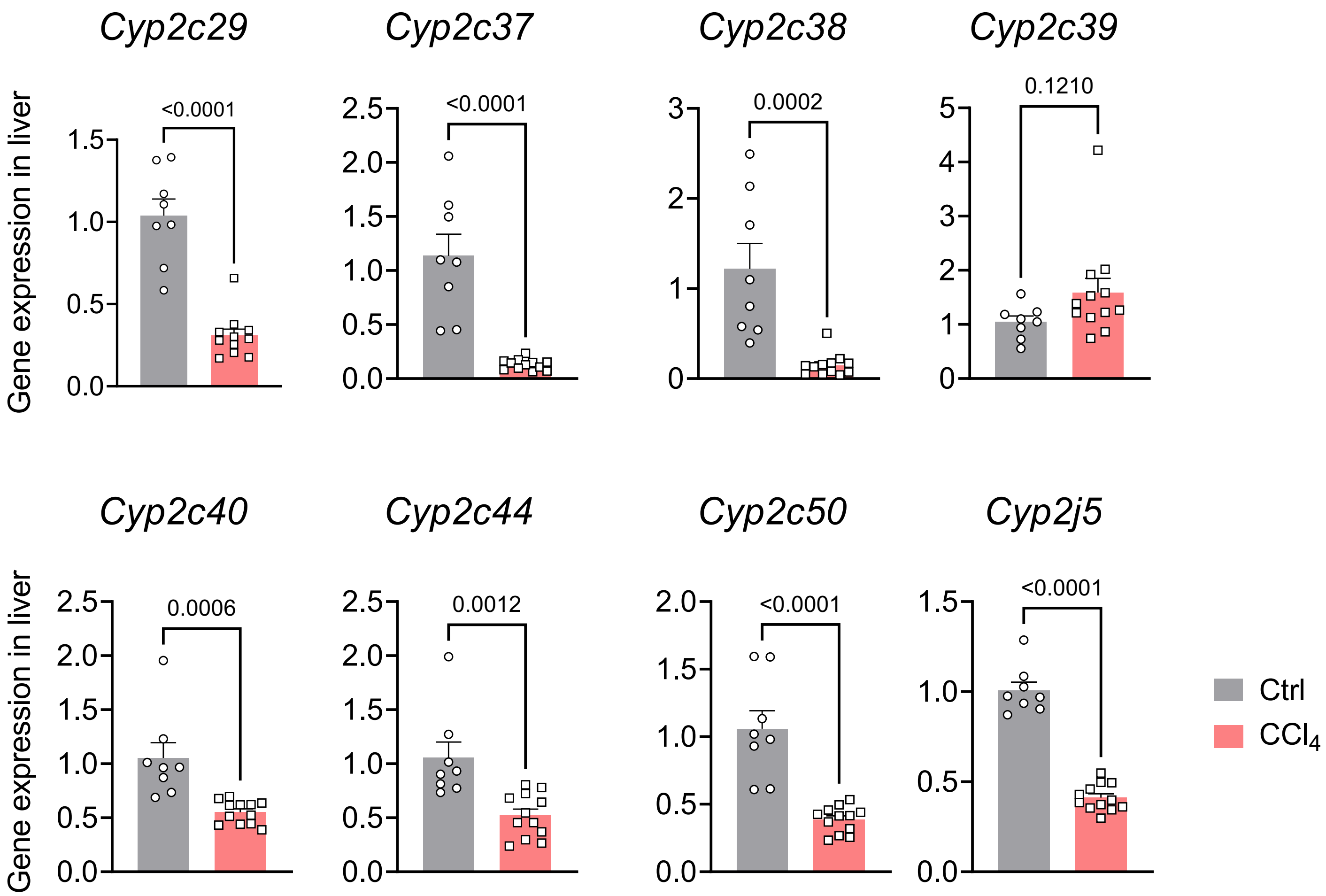

**Fig. S9. $CCl_4$ treatment reduces gene expression of *Cyp2c/2j* monooxygenases in the liver.** The data are mean ± SEM, n = 8-12 mice per group.

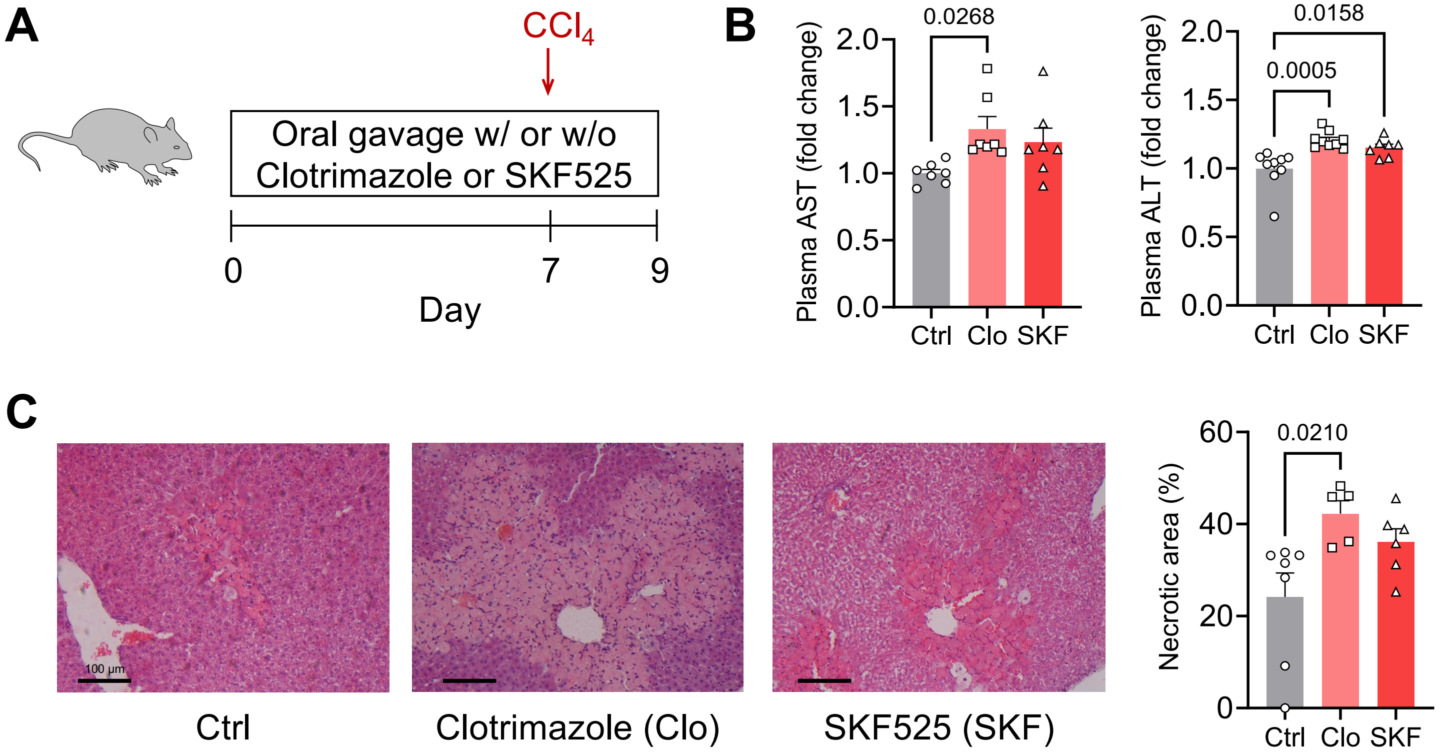

**Fig. S10. Pharmacological inhibition of CYP monooxygenases exacerbates chemically induced liver injury in mice. A.** We treated SPF C57BL/6 mice with CYP inhibitors, SKF-525A or clotrimazole, then stimulated the mice with $CCl_4$ to induce liver injury. **B.** Treatment with CYP inhibitors increases plasma concentrations of AST and ALT in mice (n = 7-9). **C.** Liver histology shows that treatment with CYP inhibitors exacerbates liver damage (n = 5-7). The data are mean ± SEM.

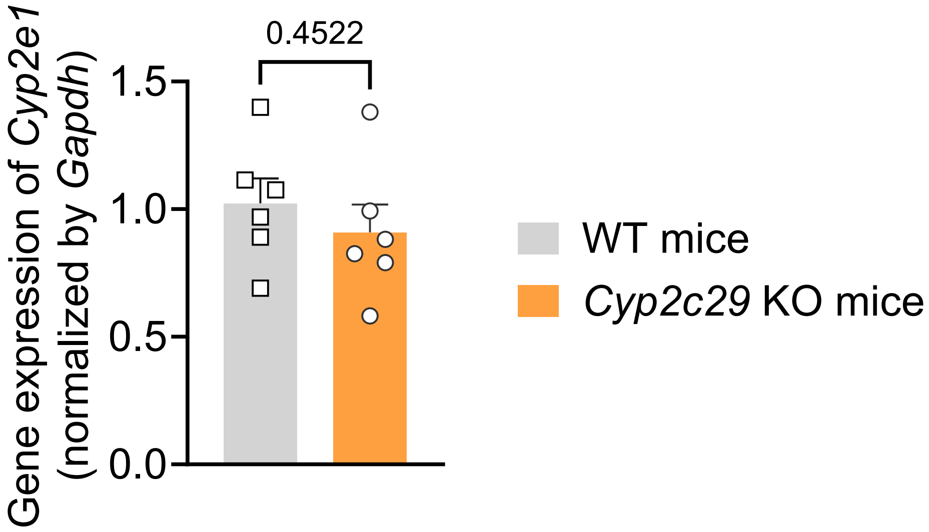

**Fig. S11.** The expression of *Cyp2e1* was not significantly changed in *Cyp2c29* KO mice compared with WT mice. The data are mean ± SEM.

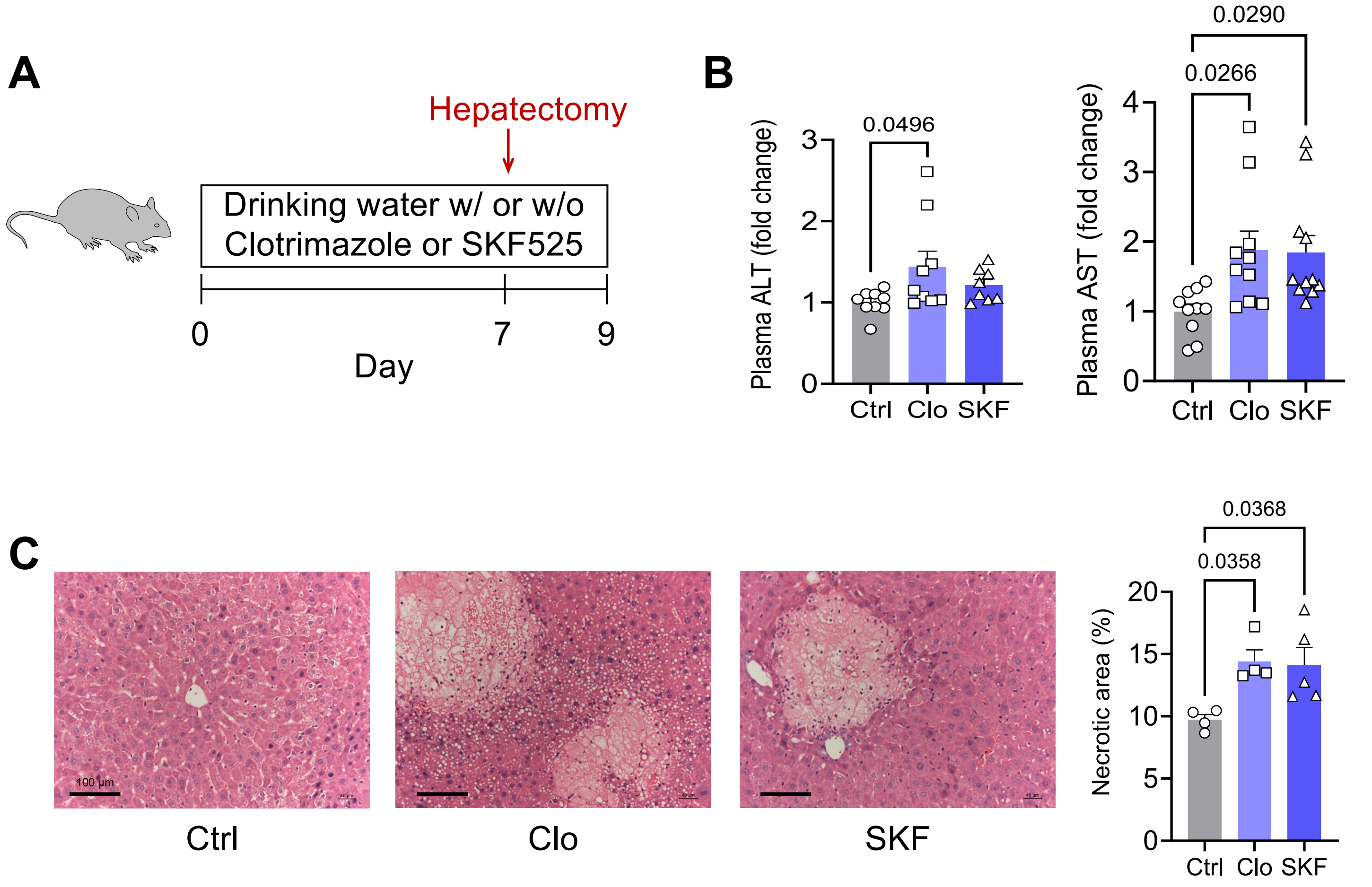

**Fig. S12. Pharmacological inhibition of CYP monooxygenases exacerbates surgically induced liver injury. A.** We treated SPF C57BL/6 mice with CYP inhibitors and then induced liver injury via hepatectomy surgery. **B.** Treatment with CYP inhibitors increases plasma concentrations of AST and ALT in mice (n = 8-11). **C.** Liver histology shows that treatment with CYP inhibitors exacerbates liver damage (n = 4-5). The data are mean ± SEM.

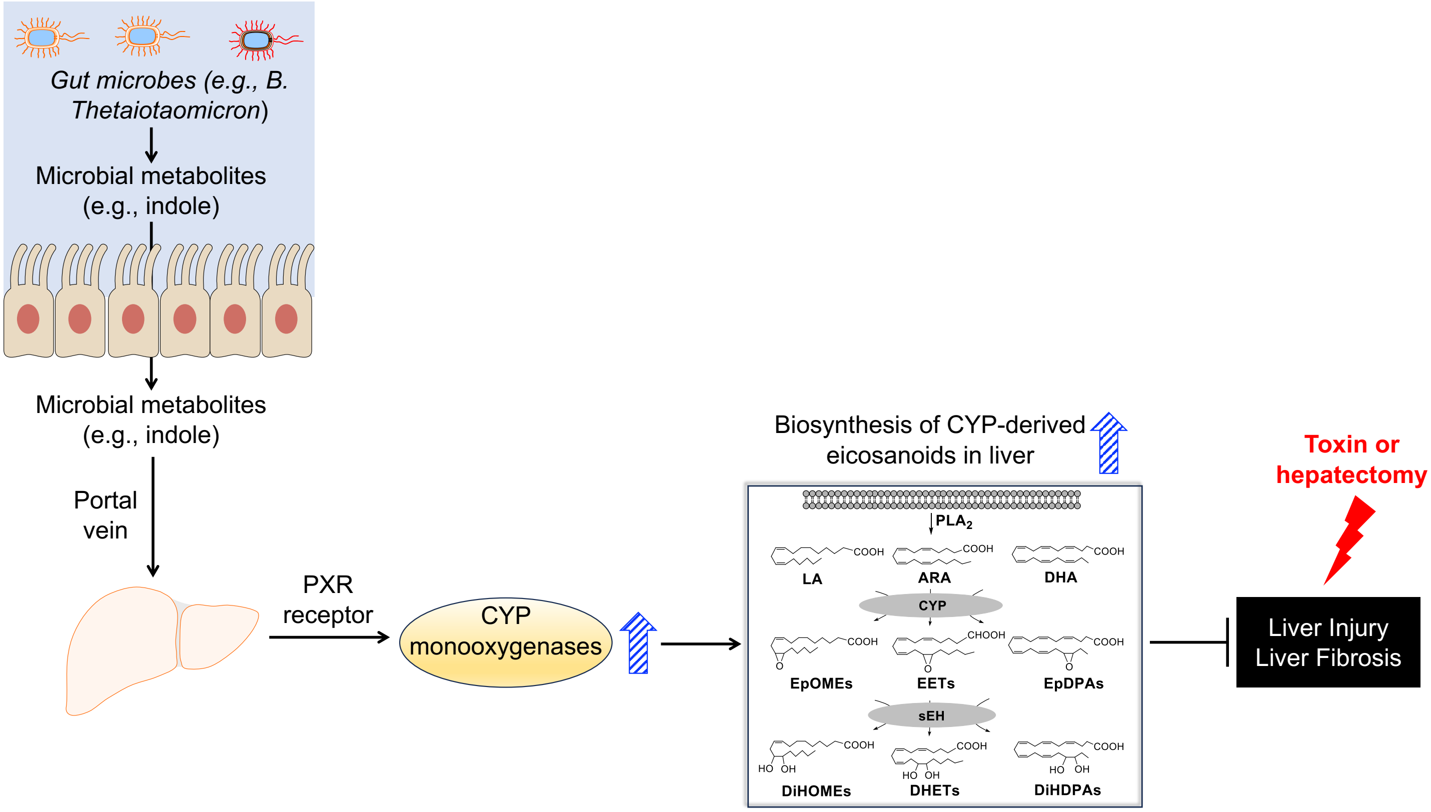

**Fig. S13. Proposed model:** Specific gut microbes, notably *B. thetaiotaomicron*, produce small-molecule metabolites such as indole. These microbial metabolites translocate from the gut to the liver via the portal vein, where they enhance the expression of CYP monooxygenases and elevate hepatic concentrations of CYP-derived eicosanoids. The upregulation of the hepatic CYP eicosanoid pathway contributes to protection against chemically induced and hepatectomy-induced liver injury and fibrosis.
